# Increasing Truck Drivers’ Compliance, Retention, and Long-Term Engagement with e-Health & Mobile Applications: A PRISMA Systematic Review

**DOI:** 10.3390/healthcare14030340

**Published:** 2026-01-29

**Authors:** Rocel Tadina, Hélène Dirix, Veerle Ross, Muhammad Wisal Khattak, An Neven, Brent Peters, Kris Brijs

**Affiliations:** 1 Transportation Research Institute (IMOB), School of Transportation Sciences, UHasselt (Hasselt University), Martelarenlaan 42, 3500 Hasselt, Belgium; roceltadina@gmail.com (R.T.); helene.dirix@uhasselt.be (H.D.); muhammadwisal.khattak@uhasselt.be (M.W.K.); an.neven@uhasselt.be (A.N.); brent.peters@uhasselt.be (B.P.); kris.brijs@uhasselt.be (K.B.); 2 FARESA, Evidence-Based Psychological Center, 3500 Hasselt, Belgium

**Keywords:** PRISMA, technology adoption, e-health, truck drivers, systematic review, persuasive design

## Abstract

**Background:** Truck drivers constitute a high-risk occupational group due to irregular schedules, prolonged sedentary work, fatigue, and limited access to healthcare, contributing to adverse physical and mental health outcomes. Although mobile health (mHealth) tools offer potential to support driver health, sustained engagement remains a persistent challenge. **Objectives:** This systematic review aimed to identify behavioural, technological, and contextual determinants influencing truck drivers’ compliance, retention, and long-term engagement with digital health interventions. **Methods:** Following the PRISMA 2020 guidelines, six eligible studies were identified and thematically synthesised across technology acceptance, behaviour change, and persuasive system design perspectives. **Results:** Across studies, sustained engagement was facilitated by self-monitoring, real-time feedback, goal-setting, coaching support, and simple, flexible system design. In contrast, technological complexity, high interaction demands, limited digital literacy, privacy concerns, misalignment with irregular schedules, and fatigue consistently undermined engagement and retention. Autonomy, trust, and voluntary participation emerged as cross-cutting determinants supporting continued use. Based on the synthesis, an integrative framework was developed to explain how behavioural, technological, and contextual factors interact to shape truck drivers’ compliance, engagement, and retention with mHealth. Despite generally moderate to high study quality, the evidence base remains fragmented and dominated by short-term evaluations. **Conclusions:** The findings highlight the importance of context-sensitive, user-centred design to support effective digital health interventions in the trucking sector.

## 1. Introduction

Truck drivers play an essential role in the logistics and transportation sector, facilitating the efficient and reliable movement of goods that sustain local economies and global supply chains [1,2]. Their contribution extends beyond freight delivery; they are indispensable to trade, commerce, and economic stability [3]. However, the profession faces a critical and worsening shortage of qualified drivers across Europe and globally [4,5]. This shortage has significant economic implications, contributing to supply-chain disruptions, rising freight costs, and delivery delays [6]. Addressing this workforce gap requires not only the recruitment of new drivers but also the retention and attention to the well-being of existing drivers, ensuring their long-term productivity and engagement in the sector.

The driver shortage is closely linked to the occupation’s demanding nature. Extended working hours, irregular schedules, prolonged sedentary periods, limited access to healthy food and healthcare, and chronic sleep deprivation contribute to widespread fatigue, obesity, cardiovascular disease, and mental health issues among truck drivers [7,8,9,10]. These health challenges are compounded by the structure of the job itself, in which drivers have no fixed workplace, spend long periods alone on the road, and have limited interaction with coworkers or supervisors. These working conditions reduce social support, strain coping capacity, reinforce unhealthy routines, and contribute to lower job satisfaction and higher turnover rate among truck drivers [9,11,12]. Cultural stigma and a prevailing “macho” work culture often discourage drivers from addressing health problems or seeking mental health support [13], further exacerbating poor health outcomes and contributing to attrition. Consequently, improving drivers’ physical and mental health is not only a matter of occupational safety and public health but also a strategic priority for addressing the European and global driver shortage.

In response to these challenges, digital health and mobile health (mHealth) interventions present promising tools to promote health, enhance safety, and strengthen retention among professional drivers. These health interventions provide accessible and scalable solutions for self-monitoring, health promotion, and fatigue management [14,15,16]. In occupational settings, such mHealth tools have been shown to improve drivers’ awareness of healthy behaviours and provide real-time feedback to support physical and mental well-being [14,16,17]. Recent advances in smartphone-based systems and digital technologies have further expanded the technical sophistication and sensing capabilities of mobile applications [18]. However, these developments often prioritise technical performance and system accuracy over sustained user engagement.

While many drivers express interest in adopting health apps, sustained engagement remains a challenge due to barriers such as digital literacy, privacy concerns, lack of personalisation, and poor alignment with real-world work conditions [19,20,21]. Many programmes remain confined to short-term behavioural prompts, such as brief reminders, one-time notifications, basic self-monitoring tasks, or simple health tips, and fail to incorporate the broader organisational and environmental stressors that shape truck drivers’ daily realities. These include organisational factors such as long shifts, irregular schedules, and workload pressure, as well as environmental constraints such as inadequate sleep facilities, limited healthy food options, and restricted access to safe parking [9,12]. Mental-health factors, including psychological stress, mental fatigue, and emotional exhaustion, are likewise underrepresented in intervention design, further limiting engagement and retention [12]. This pattern is further supported by recent adoption research demonstrating that technology adoption is shaped by the interaction of technological, organisational, environmental, and human factors, and that models focusing on technology alone systematically fail to explain sustained use in applied contexts [22]. As a consequence, without consistent engagement, even well-designed digital interventions fail to produce meaningful behavioural change [23].

As engagement is central to the challenges described above, this review clarifies how three related concepts are defined and used throughout the analysis. Compliance refers to the extent to which users follow recommended activities or protocols within digital interventions; retention denotes continued participation throughout the intervention or study period [24]; and engagement encompasses active, sustained interaction, including both behavioural and emotional involvement [25]. These conceptual definitions guided study selection, synthesis, and interpretation.

## 2. Study Objectives

This study aims to identify determinants at different levels (i.e., behavioural, technological, and contextual) that influence truck drivers’ compliance, retention, and long-term engagement with e-health and mobile applications in the transportation sector, using the PRISMA 2020 protocol. Additionally, it integrates these determinants within a theory-informed framework that connects empirical findings to established models of technology acceptance, behaviour change, and persuasive system design. This integrated approach provides both a descriptive understanding of factors influencing engagement and a conceptual foundation for developing sustainable digital-health interventions tailored to the occupational realities of professional drivers, ultimately enhancing health outcomes, job satisfaction, retention, and workforce sustainability [23,26].

Specifically, the study addresses the following research questions:

1. What factors highly influence truck drivers’ compliance, retention, and engagement with e-health and mobile applications in the transportation sector?
2. How do user demographics, preferences, and needs affect mobile application adoption and usage patterns?
3. What are the key barriers and challenges that prevent users from accepting e-health and mobile applications in the transportation sector?
  3.1 How do these barriers and challenges differ across subgroups of truck drivers (e.g., age, experience, digital literacy, and driving context)?
4. How can technology design features and system usability be leveraged to improve long-term engagement with e-health and mobile applications among truck drivers?

To support the interpretation of these research questions, this study employs multiple theoretical perspectives to guide the analysis of determinants at behavioural, technological, and contextual levels. These include technology-acceptance models such as the Unified Theory of Acceptance and Use of Technology 2 (UTAUT2) [27] and the Multi-Level Model on Automated Vehicle Acceptance (MAVA) [28]; behaviour-change theories such as Self-Determination Theory (SDT) [29,30,31,32], the Capability–Opportunity–Motivation Behaviour Model (COM-B) [33], and the Health Belief Model (HBM) [34,35,36]; and the Persuasive Systems Design (PSD) [37] framework. Table 1 summarises the frameworks and outlines their relevance to the study context.

These frameworks are used as interpretive lenses to organise, contextualise, and explain how behavioural, motivational, and technology design-related factors influence engagement, compliance, and retention in digital health contexts. For clarity, all theoretical constructs referenced in this review are defined in Appendix A.

## 3. Methodology

This study employed a systematic literature review to identify and synthesise determinants at the behavioural, technological, and contextual levels that influence truck drivers’ compliance, retention, and engagement with e-health and mobile applications. A systematic review was chosen over scoping or narrative approaches to ensure a structured, transparent, and replicable process for locating, evaluating, and integrating evidence [38,39]. Whereas scoping reviews broadly map a field without appraising study quality [40,41,42], the systematic review method ensures that the analysis is built on high-quality, peer-reviewed evidence, as it is widely recognised as the highest standard for evidence synthesis [43,44].

This systematic review was conducted and reported in accordance with the Preferred Reporting Items for Systematic Reviews and Meta-Analyses (PRISMA) 2020 protocol [45,46]. The completed PRISMA 2020 checklist is provided in Supplementary Materials, and the PRISMA flow diagram summarising the study selection process is presented in the Results.

A comprehensive search was conducted in PubMed, Scopus, Web of Science, and TRID, databases selected for their coverage of biomedical, multidisciplinary, and transportation research [47,48,49,50,51,52]. The search strategy was structured around the following key concepts: compliance, retention, user engagement, e-health, transportation sector, and truck drivers. The complete search string applied across all databases was:

(compliance OR conformity OR observance OR commitment OR retention OR continuation OR engagement OR participation OR involvement OR motivation OR user adoption)
AND
(e-health OR digital health OR telemedicine OR mHealth OR online health OR virtual health OR electronic health)
AND
(transportation sector OR transportation OR transport sector OR transport OR mobility OR transit OR traffic systems OR traffic)
AND
((truck OR heavy vehicle OR freight OR commercial OR long-haul OR professional OR logistics OR delivery) AND (driver* OR operator*))

Eligibility criteria required that studies:

- examined digital health interventions (e-health, mHealth, or telemedicine) involving truck drivers within the transportation sector;
- reported outcomes related to compliance, engagement, or retention; and
- were peer-reviewed, empirical articles published in English.

Conference papers, book chapters, editorials, retracted articles, and studies unrelated to digital health or truck driving contexts were excluded.

The database search was completed in February 2025, and all screening and data extraction activities were finalised in May 2025.

All records were exported to Zotero (Falls Church, VA, USA) for deduplication and organisation; subsequent title/abstract and full-text screening were managed in a customised Excel (Redmond, WA, USA) workbook designed to maintain a transparent, traceable workflow [53]. Screening decisions were recorded systematically with standardised reasons for exclusion at each phase (aligned to PRISMA 2020). As this review was conducted by a single primary screener, cases of uncertainty were flagged and resolved through consultation with secondary reviewers to ensure consistency and reduce subjective bias.

For each included study, a structured data matrix captured study design, setting, population, intervention type, digital platform, theoretical basis, outcomes, and key findings, alongside ethical-approval status and funding information reported by the original authors.

Critical appraisal tools are structured checklists used to assess the methodological quality, credibility, and potential bias of research studies by evaluating their design, data collection, and strategies for reducing bias [54,55]. Quality appraisal used design-appropriate tools: the Critical Appraisal Skills Programme (CASP) checklists for quantitative and qualitative studies [56,57] and the Mixed Methods Appraisal Tool (MMAT) for mixed-methods designs [58,59]. Studies were rated high, moderate, or low quality following established appraisal schemes. In line with the review’s aim to provide comprehensive coverage, no studies were excluded based on quality. Instead, appraisal ratings informed the interpretation and weighting of evidence during synthesis. As with screening, quality ratings were completed by the primary reviewer, with clarification sought from secondary reviewers in cases of doubt.

Interpretation was guided by the research questions and theoretical frameworks (UTAUT2, MAVA, COM-B, SDT, HBM, and PSD), which were used as interpretive lenses to relate empirical determinants to established constructs in technology acceptance, behaviour change, and persuasive system design.

Since this review synthesised previously published studies, no ethical approval was required. All data supporting the review are derived from the published literature; the extraction matrix and screening log are available from the corresponding author upon request.

To enhance methodological transparency, two aspects of the review process are explicitly acknowledged. First, the review was not prospectively registered. Second, study selection and data extraction were primarily undertaken by a single reviewer. Although registration and dual review are recommended best practices, they are not mandatory requirements of PRISMA 2020, and these aspects were addressed through careful supervision and transparency.

Importantly, the review was conducted under the close supervision of academic staff with methodological expertise. The review question, eligibility criteria, and analytic approach were predefined collaboratively by the reviewer and supervising academics prior to screening, and these criteria were operationalised using explicit decision rules. Conservative inclusion thresholds were applied when eligibility was uncertain, and a Supplementary Material detailing these decision rules is available. The absence of prospective registration and dual independent review is explicitly acknowledged as a limitation, and the findings are interpreted cautiously.

## 4. Results

### 4.1. Study Selection

**Figure 1:**
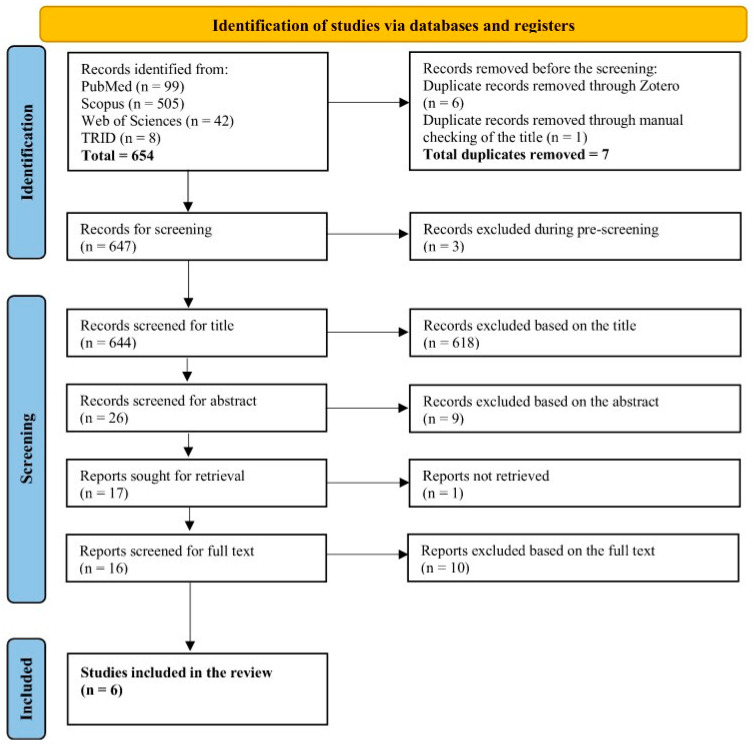
PRISMA 2020 flow diagram for study selection on truck drivers’ compliance, retention, and long-term engagement with e-health and mobile applications.

A total of 654 records were identified through database searches. After removing seven duplicates via Zotero and manual checking, 647 records were screened by title and abstract, resulting in 17 articles sought for retrieval. One article could not be retrieved, leaving 16 studies for full-text eligibility assessment, during which ethical considerations were also reviewed. Of these, 10 studies were excluded for irrelevance to the population, intervention, or outcomes. A final set of six studies was included in this systematic review, and their quality was appraised to enhance methodological transparency. The PRISMA 2020 flow diagram, as shown in Figure 1, summarises the study identification and selection process.

A detailed analysis of exclusion patterns showed that most removals fell under the combined categories E1, E2, and E3 (n = 402), representing studies that did not meet core eligibility criteria related to population, sector, or intervention type. The second largest group, E1 + E3 (n = 138), included studies involving non-truck-driver populations or lacking a digital-health component. Single-category exclusions highlighted similar trends: E1 (n = 21) for population mismatch, E2 (n = 2) for studies focused on other transport sectors such as aviation or maritime, and E3 (n = 35) for interventions without an e-health component. Smaller numbers were excluded for basic ineligibility (E0, n = 3) or insufficient access to full text (E6, n = 1). Additional exclusions at full text corresponded to outcome misalignment (E4–E5) or unsuitable publication types (E7–E9). A complete list of exclusion categories and definitions is provided in Appendix B.

### 4.2. Characteristics of Included Studies

The included studies, published between 2016 and 2022, were conducted in the United States, Canada, and the United Kingdom. They employed quantitative (n = 3), qualitative (n = 1), and mixed-methods (n = 2) designs, reflecting varied methodological approaches to evaluating digital health interventions among professional truck drivers. Intervention types included mobile health coaching programmes, fatigue-monitoring and self-tracking tools, and mobile applications promoting physical activity and dietary improvement. Outcomes measured across studies focused on user compliance, engagement, and retention, alongside secondary indicators such as behavioural change, fatigue reduction, and health awareness. While only two studies explicitly referenced theoretical frameworks, most reported findings consistent with constructs from behaviour change, technology acceptance, and persuasive design theories. Overall, five studies were rated high quality and one moderate, reflecting a generally strong evidence base for synthesis. The characteristics of the included studies are summarised in Table 2.

In addition to describing the characteristics of the included studies, this review identifies the key research gaps within each study to clarify why existing digital health interventions for occupational truck drivers remain limited in addressing long-term compliance, retention, and sustained engagement. These gaps, as summarised in Table 3, highlight limitations in theoretical grounding, longitudinal evaluation, and occupational specificity, thereby supporting the need for the integrated synthesis and framework developed in this research.

### 4.3. Thematic Synthesis of Determinants

Thematic synthesis was conducted to identify, interpret, and integrate determinants of engagement, compliance, and retention across the included studies. The process involved sequential mapping of study findings to the predefined research questions and to the theoretical frameworks guiding this review, followed by a cross-study synthesis of determinants. The results of these analyses informed the development of the integrative framework presented in Section 4.3.4.

#### 4.3.1. Mapping of Included Studies to Research Questions

Building on the characteristics of the included studies, each study’s contributions to the five predefined research questions are mapped in the current section, enabling a structured synthesis of evidence across key thematic areas. This mapping forms the analytical basis for cross-study comparison and the development of broader themes discussed in the following sections.

The mapping clarifies how each study contributes to the five research questions, capturing determinants related to engagement, compliance, and retention (RQ1); user demographics, preferences, and needs (RQ2); barriers to technology acceptance (RQ3); subgroup and contextual variations (RQ3.1); and the influence of technology design features on long-term use (RQ4).

Findings were interpreted in context according to study design and scope, especially where direct responses to the research questions were not available. Table 4 summarises this mapping and provides an overview of the determinants identified per theme, organised according to the corresponding research questions.

#### 4.3.2. Mapping of Included Studies to Theoretical Frameworks

This section maps the synthesised findings from the included studies to the theoretical frameworks introduced in Section 2. The mapping illustrates how truck drivers’ engagement patterns, motivational factors, perceived barriers, and interactions with digital tools correspond to key theoretical constructs, thereby strengthening the interpretation of results and informing theory-based recommendations for digital health intervention design.

Each framework contributes distinct yet complementary insights across the research questions. UTAUT2 relates primarily to user demographics, expectations, and facilitating conditions influencing adoption; MAVA contextualises organisational and trust-related factors; SDT and COM-B explain motivational and behavioural mechanisms sustaining engagement; HBM captures perceptions of health risks, benefits, and barriers; and PSD links persuasive and design features to sustained digital health use.

Table 5 summarises the alignment of findings with the six theoretical frameworks based on the key constructs reflected in the study data.

Table 5 shows that empirical findings from the included studies align most strongly with constructs from UTAUT2, SDT, and COM-B, particularly those related to perceived usefulness, ease of use, motivation, capability, and opportunity. These constructs were reflected across multiple studies and contexts, indicating their central role in explaining both adoption and sustained engagement with digital health tools among truck drivers.

Constructs associated with MAVA and HBM were identified less consistently and were primarily reflected through contextual and perceptual factors, such as trust in automation, perceived control, perceived health risks, and perceived barriers. These constructs tended to emerge indirectly through reported concerns about monitoring, autonomy, fatigue, and work-related constraints rather than through explicit measurement.

Alignment with the PSD model was observed mainly in relation to primary task support, dialogue support, and social support features, while system credibility support was not directly reflected in the study findings.

#### 4.3.3. Cross-Study Summary of Determinants Identified Through Mapping

To integrate evidence across all six included studies, this section consolidates the determinants that influenced truck drivers’ compliance, retention, and engagement with e-health and mobile applications. These determinants were derived from the thematic mappings presented earlier, covering engagement mechanisms, user characteristics, contextual barriers, and technological features, and represent the recurring behavioural and environmental factors shaping user interaction with digital health tools.

In total, 15 core determinants were identified, encompassing behavioural-level motivators (e.g., self-monitoring, feedback, personalisation), technological enablers (e.g., usability, access, perceived usefulness), and contextual constraints and enablers (e.g., work schedules, privacy concerns). Table 6 summarises these determinants, providing concise descriptions and indicating which studies reported supporting evidence.

This synthesis provides a comprehensive foundation for understanding the multilevel drivers of digital health engagement among professional drivers and serves as the basis for the integrative framework presented in the following section.

#### 4.3.4. Integrative Framework Linking Theory and Empirical Determinants

This section synthesises constructs from six theoretical frameworks (i.e., UTAUT2, MAVA, SDT, COM-B, HBM, PSD) into four functional domains that collectively explain behavioural compliance, retention, and long-term engagement with digital health tools among truck drivers. The framework provides an integrated view of how individual beliefs, motivational processes, contextual factors, and design features interact to influence sustained technology use.

The four domains are summarised below:

**1.**  **Individual Beliefs and Perceptions**

This domain captures the cognitive evaluations that shape initial openness toward digital health tools. Constructs such as performance expectancy, perceived benefits, and perceived barriers explain how drivers assess whether an intervention is worth adopting given their health needs, work demands, and perceived risks. In the reviewed studies, perceived usefulness and relevance to driving routines were decisive, whereas abstract health benefits or generic wellness messaging were insufficient. This domain, therefore, explains why drivers may initially accept or reject a health technology based on internal judgments and perceived needs, but it does not account for sustained use on its own.

Key constructs: UTAUT2/MAVA—Performance and effort expectancy; UTAUT2—Price value; MAVA—Safety and service of technology, travel behaviour, socio-demographic, personality traits; HBM/MAVA—Perceived susceptibility/risks, perceived benefits; HBM—Perceived barriers, perceived severity, self-efficacy.

(Note: Perceived severity is retained conceptually because it represents potential influences on adoption (i.e., fear of illness or preventive motivation) but was not directly associated with modifiable digital design factors or measurable determinants in the reviewed studies.).

**2.**  **Motivational and Psychological Drivers**

Sustained engagement is primarily explained through this domain, which reflects the internal processes that maintain behaviour over time. Drawing on SDT and COM-B, this domain emphasises autonomy, competence, relatedness, and motivational regulation as central to long-term engagement. The framework highlights that engagement declines when drivers experience loss of control, excessive monitoring, or low confidence in using the technology, even if initial adoption occurs. Conversely, voluntary participation, self-paced use, and meaningful feedback support intrinsic motivation and habit formation. This domain explains what drives sustained behavioural action and user commitment, particularly in demanding work environments such as long-haul trucking.

Key constructs: SDT—Autonomy, competence, and relatedness; COM-B—automatic/reflective motivation; UTAUT2/MAVA—Hedonic motivation; UTAUT2—Habit.

**3.**  **Contextual Enablers and Barriers**

This domain reflects external and environmental factors that enable or constrain technology use in real-world conditions and situates individual motivation within the occupational reality of trucking. Facilitating conditions, opportunity structures, and cues to action are shaped by work schedules, fatigue, regulatory requirements, organisational culture, and access to infrastructure. The framework demonstrates that motivation and capability are conditional on context: even highly motivated drivers disengage when tools conflict with shift patterns, require frequent interaction, or lack offline functionality. This domain answers the question: Under what circumstances can engagement occur, and what structural conditions hinder or support it?

Key constructs: UTAUT2/MAVA—Facilitating conditions, social influence; COM-B—Physical/psychological capability, physical/social opportunity; MAVA—Exposure to technology; HBM—Cues to action.

**4.**  **Design Features That Promote Engagement**

This domain relates to system-level features and persuasive mechanisms built into the technology to encourage usage and interaction. Persuasive design mechanisms such as primary task support, dialogue support, system credibility support, and social support function as behavioural triggers that activate and reinforce motivation across time. Importantly, the framework positions design not as an independent driver but as a mediating layer that operationalises psychological and contextual determinants. For instance, features such as simplicity and adaptive feedback reduce cognitive load and support engagement under fatigue and time pressure. This domain answers how system design can nudge or sustain behaviour change through motivational triggers.

Key constructs: PSD—Dialogue support, primary task support, social support, system credibility support.

(Note: System credibility support remains conceptually represented but unlinked to empirical determinants, as none of the included studies directly assessed perceptions of credibility or trust in the digital platform itself.).

**Figure 2:**
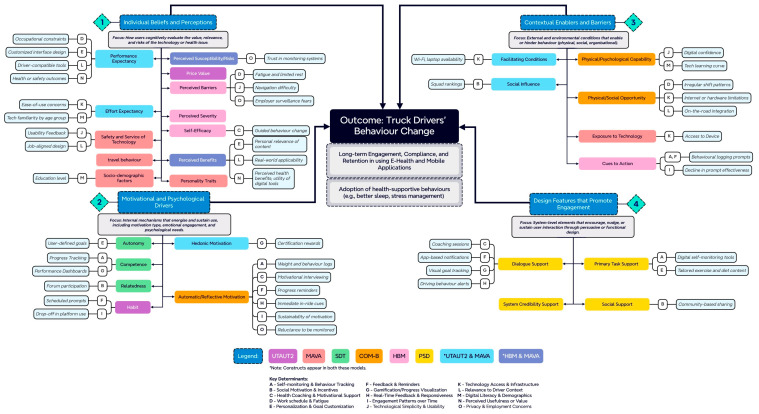
Integrated conceptual framework linking theoretical frameworks and empirical determinants influencing truck drivers’ behaviour on long-term engagement, compliance, and retention in using e-health and mobile applications.

The framework, as shown in Figure 2, serves two main purposes. First, it provides a unified perspective for understanding the behavioural processes that underlie user engagement with digital health tools. Second, it integrates these theoretical constructs with empirically derived determinants from the systematic review, bridging theory and real-world evidence from occupational health and transportation contexts. Together, the framework demonstrates how individual beliefs, motivational drivers, contextual enablers, and persuasive design features interact to shape compliance, retention, and sustained engagement with digital interventions in the transportation sector.

## 5. Discussion

### 5.1. Study Quality, Characteristics, and Gaps in the Literature

The screening and inclusion process revealed critical insights into the state of digital health research for truck drivers. A large proportion of excluded studies fell under categories reflecting a lack of integration between health, technology, and transportation. Many addressed these domains independently, but not in a combined occupational framework, echoing findings that digital health interventions for mobile or logistically complex workforces remain underdeveloped [16,65]. These patterns highlight a persistent lack of occupationally specific digital health research focused on the target population, which is truck drivers.

The scarcity of digital health interventions tailored to truck drivers reinforces earlier evidence that the transport sector lags behind others, such as manufacturing or healthcare, in adopting mobile and connected health technologies [66,67,68]. Structural and logistical challenges, including the mobile nature of work, long hours, and limited access to digital infrastructure, continue to constrain innovation and implementation [66,69].

Several excluded studies also met the population and intervention criteria but did not focus on behavioural or implementation outcomes such as compliance, retention, or long-term engagement. Valentine et al. (2025) [23] noted a similar limitation in their meta-analysis, where studies frequently reported app efficacy but omitted sustained use metrics, which are critical for real-world adoption. These exclusions highlight an ongoing tendency in the literature to evaluate health technologies in terms of clinical outcomes while overlooking user behaviour and engagement in applied occupational settings.

Among the included studies, methodological diversity and limited theoretical grounding further restricted comparability and generalisability. Most research was conducted in North America and Western Europe, focusing on long-haul or freight drivers, mirroring the regional concentration reported in previous digital health reviews [17,65]. Quantitative designs such as those by Wipfli et al. (2019) [60], Levi-Bliech et al. (2019) [62], and Heaton et al. (2017) [61] provided structured outcome data but lacked contextual richness. In contrast, qualitative approaches like Greenfield et al. (2016) [14] and Versteeg et al. (2018) [64] captured detailed insights into user perceptions and workplace realities but were limited by small, non-representative samples. This aligns with observations by Valentine et al. (2025) [23], who argued that both qualitative and quantitative limitations contribute to underdeveloped design practices in persuasive digital health tools.

Relatively few studies employed mixed-methods designs, and even fewer grounded their interventions in established theoretical frameworks such as COM-B, UTAUT2, or SDT. This lack of theoretical grounding limits the interpretability of outcomes and weakens the capacity to generalise behavioural mechanisms. Similar critiques were raised by Olson et al. (2016) [67] and Virgara et al. (2024) [68], who emphasised the need for more theory-based, participatory approaches in health interventions targeting mobile populations. Wipfli et al. (2019) [60] was the only study implementing a multi-component intervention specifically tailored to truck drivers, highlighting a continued lack of purpose-built solutions that align with occupational constraints. Most other studies did not monitor user engagement beyond pilot testing, and while none explicitly stated the reason for this absence of long-term follow-up, their short study durations and single-phase designs suggest methodological or logistical constraints that limited extended evaluation [23,70]. Evidence from other occupational health contexts supports this interpretation, as limited funding cycles, compressed project timelines, and the difficulty of tracking mobile workforces often restrict the feasibility of long-term follow-up [67,68].

Finally, digital literacy and access to infrastructure were often mentioned as influencing factors but were not directly measured in most studies. Although this represents a methodological gap, it also reflects the early stage of digital health integration within the trucking industry. Callefi et al. (2022) [66] similarly noted that infrastructure readiness varies widely across regions and organisations, complicating standardised measurement efforts. However, the repeated mention of these variables, even if unreliable, highlights their perceived importance and suggests promising directions for more targeted measurement in future research.

Overall, the heterogeneity in study designs, the limited use of behavioural theory, and the scarcity of longitudinal data point to important directions for future research. A shift toward more integrated, user-centred, and longitudinal designs could enhance both the theoretical accuracy and practical relevance of digital health interventions for transport-sector populations.

### 5.2. Discussion of Synthesised Results

#### 5.2.1. Determinants of Compliance, Retention, and Engagement in Context

The most frequently reported facilitators of engagement, including self-monitoring, feedback, and personalisation, align with behaviour change strategies shown to be effective in mobile health studies. Valentine et al. (2025) [23] identified self-monitoring and real-time feedback as persuasive design elements that significantly enhanced short-term engagement in digital health applications, although their long-term impact was limited without sustained motivational reinforcement. Similarly, this review found that initial compliance often declined not only because of occupational fatigue, irregular schedules, and limited recovery time on the road but also due to competing non-work demands that drivers must balance alongside participation in an intervention.

While behaviour change strategies such as coaching and self-monitoring produced moderate to large effects in weight loss interventions among truck drivers, dropout rates remained high because most programmes did not accommodate the mobile and time-constrained nature of drivers’ work [71]. A similar pattern was seen in the SHIFT programme [67], which achieved significant reductions in body mass index but experienced attrition rates exceeding 40%. The study did not include long-term follow-up beyond the programme period, so it remains unclear whether behavioural improvements were maintained or whether relapse occurred after the intervention ended. These limitations reinforce that interventions must align with drivers’ work conditions, such as long hours, limited rest opportunities, and fluctuating schedules, to maintain engagement beyond the initial phase.

Contextual barriers, including fatigue, time scarcity, and limited digital literacy, also disrupted ongoing participation. These findings correspond with Ng et al. (2015) [16], who noted that even well-designed interventions failed to sustain behavioural change among truck drivers unless occupational stressors and environmental constraints were directly addressed.

Autonomy, competence, and relatedness, the core constructs of SDT, were also central to drivers’ motivational engagement. Versteeg et al. (2018) [64] and Crizzle et al. (2022) [63] found that drivers were more likely to continue using digital health applications when they felt in control of their health decisions and confident in managing the technology. This supports Valentine et al. (2025) [23], who emphasised that persuasive design features alone are insufficient and that digital health tools must also meet users’ psychological needs to sustain motivation and long-term retention.

#### 5.2.2. Occupational Constraints as Engagement Barriers

A range of work-related and environmental pressures limits drivers’ sustained engagement with digital health tools. Fatigue, limited digital literacy, and restricted time, which are conditions common in long-haul operations, emerged as persistent barriers across the included studies. These constraints are reinforced by the findings of de Winter et al. (2024) [70], who reported that Dutch truck drivers face considerable health-related challenges linked to their work environment, particularly chronic fatigue and restricted access to healthy food or exercise facilities. Similarly, Garbarino et al. (2018) [8] demonstrated a strong association between poor sleep hygiene, mental health issues, and disengagement from health-related behaviour change. These findings correspond to behavioural barriers outlined in the COM-B model, particularly reduced physical and psychological capability.

Additional evidence on occupational engagement barriers comes from a naturalistic trial that tested heart-rate-based drowsiness monitoring devices [72]. Although the wearable technology led to a measurable reduction in harsh braking events, its sensitivity in predicting real-time fatigue was limited. In addition, behavioural adjustments among drivers appeared to result more from the presence of the monitoring device than from the alerts themselves. This highlights the influence of perceived oversight and the psychosocial environment in shaping health-related behaviours among truck drivers.

#### 5.2.3. User Readiness, Technology Simplicity, and Usability

Technological simplicity emerged as a cross-cutting determinant of engagement, particularly among older or less digitally literate drivers. These users were frequently excluded from more complex tools, reflecting patterns observed in developing-country mHealth deployments, where demographic factors limited adoption even when tools were technically accessible. Simplicity and perceived ease of use appear to be prerequisites for successful adoption, especially for users with limited digital skills.

Callefi et al. (2022) [66] offered a broader systems-level lens, describing 32 technology-enabled capabilities such as automation, real-time sensing, and connected monitoring systems that have the potential to transform freight transportation. In this study’s framework, these capabilities are not individual health interventions but sector-wide technologies with potential relevance for safety, efficiency, and well-being. They emphasised that actual implementation is constrained by varying levels of user readiness and contextual feasibility. Many of the high-readiness technologies, such as real-time health or vehicle monitoring, were not employed in the behavioural studies reviewed. This gap between technological availability and adoption suggests that tools must be better aligned with drivers’ competencies, motivational states, and the availability of organisational support. These findings support the synthesised results, which identified system usability and trust as critical engagement levers.

This aligns with findings that tools requiring frequent interaction or multitasking were generally less successful, even when they offered valuable health insights. For digital health interventions to be effective in trucking environments, usability must be adapted to both the cognitive load and ergonomic constraints faced by drivers.

Additionally, interventions that allowed flexible use, brief interactions, or integration into existing work systems, such as electronic logging devices (ELDs), achieved higher compliance rates. This reflects the conclusions of Hoque et al. (2020) [65], which emphasised that mHealth success in low-resource or high-demand contexts depends on simplicity, offline functionality, and minimal user input.

#### 5.2.4. Social Identity and Autonomy in Health Interventions

A critical insight from Virgara et al. (2024) [68] concerns the framing of health interventions. Programmes that emphasise health deficits, such as weight or fatigue, can inadvertently stigmatise drivers and undermine their motivation to engage. The synthesised findings of this review, particularly those relating to autonomy, competence, and relatedness, align closely with this perspective. When drivers perceive themselves as being in control of their health and capable of using the intervention effectively, they are more likely to participate. In contrast, interventions perceived as employer-enforced or punitive, such as those involving surveillance features or mandatory check-ins, may adopt resistance and distrust.

These observations further support the relevance of SDT within the integrative framework. By focusing on intrinsic motivational drivers, rather than relying on external control mechanisms, digital health interventions can more effectively encourage sustained behavioural engagement.

#### 5.2.5. Organisational and Policy-Level Influence

Beyond individual determinants, the synthesised findings highlight the critical role of organisational culture and regulatory frameworks in shaping the success of digital health interventions. Rathore et al. (2022) [69] identified several barriers to the adoption of digital innovations in freight companies, including fear of surveillance, lack of managerial support, and ambiguous data governance structures. These organisational challenges help explain why even well-designed health applications often face implementation difficulties. When digital interventions are perceived as tools for driver surveillance rather than as resources for personal benefit, drivers may disengage. This supports the theoretical implication that interventions perceived as intrusive or authoritarian can weaken relatedness and trust, thereby reducing retention.

Similarly, Callefi et al. (2022) [66] emphasised that the deployment of technology-enabled capabilities is often constrained not by the technologies themselves, but by institutional inertia, unclear policies, and fragmented decision-making within the freight sector. The synthesis of theoretical models in this study offers a useful lens through which to interpret these systemic barriers, suggesting that effective digital health strategies must extend beyond user-centred design to include coordinated efforts across multiple stakeholders.

#### 5.2.6. Theoretical Coherence of the Integrated Framework

The integrated framework presented in Section 4.3.4 demonstrates that combining theoretical models allows for a nuanced interpretation of behavioural determinants. For example, the self-monitoring determinant aligns with PSD’s element of “primary task support” and simultaneously reflects cues to action from UTAUT2, automatic and reflective motivation from COM-B, and competence from SDT. Similarly, gamification and social incentives address hedonic motivation in UTAUT2 and the need for relatedness in SDT, respectively, reinforcing engagement through enjoyment and social connection.

However, several constructs included in the theoretical models, such as system credibility support, travel behaviour, and perceived severity, were not supported by evidence in the studies reviewed. This observation highlights a gap between theoretical frameworks and practical applications. It suggests that future research and intervention design should empirically assess the relevance of less frequently supported constructs before incorporating them into design guidelines.

Crucially, the framework emphasises that long-term engagement emerges from the interaction of all four domains, rather than from any single determinant. Individual beliefs influence initial adoption, motivation sustains behaviour, context constrains or enables use, and design features operationalise engagement strategies. This interactional structure explains why interventions that focus solely on persuasive features or behaviour change techniques often fail when occupational constraints are ignored.

### 5.3. Contextualising Advanced and Emerging Digital Health Systems

Recent advances in digital health technologies have expanded the range of tools available to support occupational health monitoring for professional truck drivers, particularly in contexts where mobility, irregular schedules, and limited access to traditional healthcare pose persistent challenges. Technologies such as wearable health devices, Digital Twin systems, telemedicine platforms, and artificial intelligence (AI)-enabled applications share a common emphasis on continuous data collection, remote monitoring, and data-driven health support, making them conceptually relevant to the engagement, compliance, and retention determinants identified in this review [73,74,75]. Rather than representing distinct or isolated solutions, these approaches can be understood as complementary components of an evolving digital health ecosystem for occupational driver populations.

Across these technologies, a central opportunity lies in their capacity to collect and integrate physiological, behavioural, and contextual data in real time. Wearable health devices and flexible sensor technologies enable unobtrusive, long-term monitoring of indicators related to fatigue, physical activity, and overall well-being, which is particularly suited to the mobile and physically demanding nature of truck driving [74,76]. When supported by Internet of Things (IoT) transmission systems and big data analytics, such data streams can be aggregated to inform personalised feedback, wellness prediction, and preventive health strategies [74,75]. Building on similar data infrastructures, Digital Twin technologies have been proposed as dynamic virtual representations that integrate sensor-driven data to simulate and monitor health states, offering more holistic and individualised insights for preventive and supportive interventions [77,78,79]. Telemedicine platforms further extend these capabilities by enabling remote consultations, follow-up care, and health monitoring, reducing barriers related to distance and time that are especially relevant for drivers with irregular schedules [73,80]. AI–enabled systems, beyond generative AI, have similarly been incorporated into digital health applications to process large and heterogeneous data streams, supporting personalisation, real-time monitoring, and predictive health analytics in mobile and remote populations [81,82].

Interpreted in light of the findings of this systematic review, the potential benefits of these advanced and emerging digital health systems are closely tied to the same engagement-related determinants identified across the included studies. While these technologies offer opportunities to support healthier lifestyles, improved health management, and preventive care for professional truck drivers, their effectiveness is unlikely to be realised through technical capability alone. Sustained engagement, perceived usefulness, user trust, transparency in data use, and compatibility with drivers’ work routines remain critical factors influencing compliance, retention, and long-term impact across wearable systems, Digital Twin approaches, telemedicine services, and AI-enabled applications [73,75,76,81]. Accordingly, these technologies are best viewed as enabling frameworks whose real-world value depends on how well they align with the behavioural, organisational, and contextual conditions shaping truck drivers’ everyday work environments.

## 6. Certainty and Strength of Evidence

The overall certainty of the evidence synthesised in this review is characterised as moderate to high. Five of the six included studies were rated as high quality, and one as moderate, based on structured appraisal using the CASP and MMATs. Despite methodological variation, the studies collectively provided credible insights into behavioural and technological determinants of engagement.

Several characteristics of the digital health evidence base require careful interpretation. Rapid technology evolution makes randomised controlled trials difficult to implement, resulting in reliance on observational or self-reported data that may introduce bias [83,84,85]. Many studies also reported only short-term outcomes, offering limited insight into the durability of behavioural change [20,25].

Considerable heterogeneity across interventions, ranging from mobile apps to wearable and web-based platforms, reduced comparability across studies [86,87]. The use of self-reported compliance and engagement measures introduces additional concerns related to recall and social desirability bias [88,89,90].

Despite this variability, the recurrence of key behavioural determinants, such as trust, ease of use, autonomy, and contextual fit, across diverse methodologies and settings supports the reliability of the synthesised findings. Although generalisability remains limited, the convergence of results across studies indicates that the core conclusions on engagement drivers among truck drivers are supported by moderately strong and trustworthy evidence, as reflected in established appraisal tools [57,59].

## 7. Limitations

Although this review followed the PRISMA 2020 framework to ensure transparency and objectivity, several limitations must be acknowledged that may affect the comprehensiveness and neutrality of the findings.

As noted in the Methodology, the review was conducted without prospective registration and with a single primary reviewer throughout the entire screening and review process. This increases the possibility of subjective judgement, particularly during full-text screening and data extraction. However, safeguards were applied to mitigate these risks. Uncertainty was not frequent, but when it occurred, decisions were discussed with secondary reviewers to verify interpretations and ensure consistency. All screening decisions and appraisal notes were systematically documented to maintain transparency and traceability throughout the review process. Published methodological guidance also recognises that single screening can be acceptable in focused or resource-constrained reviews when supported by verification steps and clear documentation [91].

Second, restricting the review to English-language, peer-reviewed articles introduces potential language and publication bias. This decision was driven by accessibility and resource-related constraints, as translation or multilingual screening was not feasible within the scope of this review. The restriction, therefore, reflects practical considerations rather than any assumption of higher quality in English-language publications [92,93].

Third, the database selection was guided by methodological relevance rather than breadth alone. PubMed, Scopus, Web of Science, and TRID were chosen because they collectively cover biomedical, multidisciplinary, and transport-specific literature, offering strong alignment with the review’s aims. While additional repositories or grey-literature archives might have identified further records, expanding the search beyond these core databases would have required substantial screening of low-yield sources, which was not feasible within the scope of this review. The selected databases, therefore, provided a balanced strategy, ensuring high relevance and manageable volume without compromising methodological rigour.

Despite these limitations, the review process was conducted using a structured, replicable, and transparently documented workflow aligned with PRISMA 2020 reporting standards [45,46]. These measures enhance confidence in the reliability of the findings while acknowledging the constraints inherent to a single-reviewer systematic review.

## 8. Future Research

From a broader perspective, this systematic review contributes to an emerging body of evidence highlighting engagement, compliance, and retention as central challenges in digital health interventions for professional truck drivers. Rather than pointing to the superiority of specific technologies or intervention types, the findings highlight the importance of behavioural, organisational, and contextual factors that shape sustained use across diverse digital health approaches. This perspective suggests that technological innovation alone is unlikely to address persistent health and retention challenges within the trucking sector unless accompanied by careful consideration of drivers’ working conditions, routines, and lived experiences.

The review also highlights the fragmented and intervention-specific nature of the current evidence base, with limited synthesis across technological, behavioural, and contextual dimensions. Viewed through this lens, the value of the existing literature lies less in demonstrating definitive effectiveness and more in revealing recurring patterns that inform how digital health tools are adopted, used, and discontinued in real-world settings. As digital health technologies continue to evolve, this perspective emphasises the need to interpret emerging solutions in relation to these underlying determinants, rather than as standalone innovations.

## 9. Recommendations

The findings of this systematic review highlight several principles to guide the design and implementation of digital health interventions for mobile and occupationally constrained populations such as truck drivers. These recommendations are intended for digital health designers, developers, practitioners, and sectoral stakeholders seeking to build context-aware and sustainable mHealth tools.

Across the six included studies, engagement and long-term retention depended strongly on how well interventions aligned with the realities of trucking work—irregular schedules, isolation, and inconsistent internet connectivity. Programmes that failed to accommodate these conditions showed lower adoption or higher dropout rates. To support sustained use, interventions should allow flexible timing, brief interactions, and offline functionality, and should avoid requiring uninterrupted attention during shifts.

Simplicity and accessibility were consistently associated with higher engagement. Applications requiring minimal user input and offering clear feedback, self-monitoring, and goal-setting, particularly when paired with motivational or coaching support, reinforced continued use [14,60,63]. To increase retention in practice, designers may incorporate:

- automated data capture rather than manual input,
- micro-interactions (e.g., 10–20 s tasks during breaks),
- adaptive reminders that adjust to trip length or driver fatigue, and
- optional check-ins instead of fixed daily tasks.

Privacy and autonomy also emerged as critical determinants of adoption, with drivers avoiding tools perceived as employer-controlled. Developers should prioritise transparent data practices, clear consent pathways, and user control over information sharing to build trust.

PSD features such as gamification, progress tracking, and reminders can enhance motivation when used sensitively to support autonomy rather than surveillance. Because digital literacy varies widely, intuitive interfaces, simple navigation, and brief onboarding are essential to lower barriers for less tech-confident users [61].

At the sectoral level, digital health tools should integrate seamlessly with work routines and existing systems, such as electronic logging devices (ELDs), to reduce friction and ensure usability [63]. Organisational policies promoting ethical data governance and user privacy protections are important to address surveillance concerns and encourage participation. Collaboration among developers, logistics companies, and policymakers can support standardised data practices and promote equitable access, particularly for independent drivers.

Future research should focus on longitudinal, theory-informed studies to better understand sustained engagement mechanisms. Comparing engagement across platforms and examining demographic or cross-cultural differences could reveal design features that drive long-term retention. Research on onboarding, training, and co-design with drivers and employers will further strengthen the relevance, usability, and scalability of digital health tools across the transport sector.

## 10. Conclusions

This systematic review aimed to identify and synthesise behavioural, technological, and contextual determinants influencing truck drivers’ compliance, retention, and long-term engagement with digital health and mobile applications. Through a systematic literature review following the PRISMA 2020 protocol, six eligible studies were identified and analysed, reflecting a small but methodologically diverse evidence base focused on occupational digital health interventions for professional drivers.

Across the included studies, sustained engagement was consistently associated with interventions that aligned with drivers’ occupational realities and supported autonomy, usability, and routine compatibility. Key determinants included self-monitoring, real-time feedback, goal-setting, coaching or motivational support, and technological simplicity. In contrast, engagement and retention were undermined by technological complexity, high interaction demands, limited digital literacy, privacy concerns, and misalignment with irregular schedules, fatigue, and constrained work environments. These findings demonstrate that engagement is shaped not by technological capability alone, but by the interaction between behavioural motivation, system design, and contextual conditions.

These insights were mapped onto an integrative framework that combined elements from the UTAUT2, MAVA, SDT, COM-B, HBM, and PSD, offering a comprehensive overview of how individual beliefs, motivational drivers, contextual enablers, and persuasive design features interact to shape compliance, retention, and sustained engagement with digital interventions in the transportation sector. The framework highlights that long-term engagement emerges from the combined effect of these domains rather than from any single intervention component.

Despite generally moderate to high study quality, the review also revealed important gaps in the literature, including limited longitudinal evaluation, inconsistent theoretical grounding, and a narrow focus on short-term outcomes. These limitations highlight the need for future digital health interventions and evaluations to better reflect the unique occupational realities of the trucking workforce and to prioritise sustained engagement as a primary outcome.

Overall, this review contributes a structured synthesis of engagement determinants and a theory-informed framework to guide the design, evaluation, and interpretation of digital health interventions for truck drivers. By emphasising contextual fit, autonomy, and usability, the findings support more realistic and sustainable approaches to improving health, well-being, and retention in the transport sector.

## Figures and Tables

**Table 1:** Theoretical frameworks and their applications to this study.

Framework		Key Concepts	Key Constructs	Applications
**Technology Acceptance Models**	**UTAUT2**	Technology acceptance based on the user expectations and external support	Performance expectancy; Effort expectancy; Social influence; Facilitating conditions; Hedonic motivation; Price Value; Habit	Explains drivers’ likelihood to adopt mobile health apps based on usability and support
	**MAVA**	Technology acceptance influenced by multi-level contextual factors; a model rooted in UTAUT-C and Car Technology Acceptance Model (CTAM)	Micro-level (individual traits, demographics personality, travel behaviour); Meso-level (exposure, domain-specific, symbolic-affective, moral-normative)	Considers external and contextual variables relevant to driver environments and routines
**Behaviour-Change Theories**	**SDT**	Motivation as intrinsic or extrinsic, shaped by psychological needs	Autonomy; Competence; Relatedness	Informs the design of interventions that promote autonomous motivation and engagement
	**COM-B**	Behaviour influenced by capability, opportunity, and motivation	Physical/psychological capability; Social/physical opportunity; Automatic/reflective motivation	Identifies barriers and enablers for behavioural change among occupational drivers
	**HBM**	Health behaviour shaped by beliefs and perceived risks	Perceived Susceptibility; Perceived Severity; Perceived Benefits; Perceived Barriers; Cues to Action; Self-efficacy	Explains health perception and readiness to adopt health interventions among drivers
**Persuasive Systems** **Design**	**PSD**	User engagement driven by persuasive system features	Primary Task Support; Dialogue Support; System Credibility Support; Social Support	Supports app design strategies that maintain long-term engagement and motivation

**Table 2:** Summary of characteristics of the included studies on digital health interventions and adoption for truck drivers.

Study	Author & Year of Publication	Country	Objective	Design	Participants	Digital Platform/Tool Used	Theoretical Framework	Main Outcomes	Quality
1	Wipfli et al. (2019) [60]	USA	To evaluate the implementation and process outcomes of the SHIFT * intervention, a mobile weight loss and health promotion programme tailored for truck drivers, with a focus on programme delivery, driver engagement, and contextual barriers/facilitators to adoption.	Quantitative (cluster-RCT)	134 overweight drivers	SHIFT web platform, cTRAIN, mobile coaching	Ecological Perspective; SCT; Operant Theory; TWH	−3.31 kg body weight; Web-based self-monitoring was the strongest predictor; Motivational interviewing improved diet	High
2	Greenfield et al. (2016) [14]	UK	To explore truck drivers’ perceptions of wearable health devices and assess how these perceptions affect their willingness to engage with health promotion technologies while on the road.	Qualitative (focus groups)	34 male drivers	Discussion on wearables (Fitbit, apps)	Psychological phenomenology approach	Openness to wearables; motivated by prevention or health-fears; privacy concerns	High
3	Heaton et al. (2017) [61]	USA	To examine how truck drivers use the internet and mobile technology, and to assess their interest in engaging with health-related interventions delivered via digital platforms.	Quantitative (survey)	106 long-haul drivers	Personal devices (laptops, phones)	None	Internet is used more for job-related tasks rather than health; Age differences affect internet usage	Moderate
4	Levi-Bliech et al. (2019) [62]	UK & USA	To investigate how mobile apps influence employee behaviour in the workplace by identifying key motivators, usage patterns, and user satisfaction, with implications for digital engagement in occupational settings.	Quantitative (observational)	11,805 trips logged from 109 fleet drivers	Fleet-management app	Feedback Theory; Experiential Learning Theory	App use decreased risky driving; mitigated by real-time notifications and amplified with app usage experience	High
5	Crizzle et al. (2022) [63]	Canada	To assess the impact of electronic logging devices (ELDs) on driver fatigue, regulatory compliance, and safety outcomes in the trucking industry following their mandatory implementation in North America.	Mixed methods	59 long-haul drivers	Electronic Logging Devices (ELDs)	Phenomenological approach (no formal theory)	ELD reduced fatigue; Improved sleep quality; Lowered stress; Concerns about reduced income, parking access, and learning curve	High
6	Versteeg et al. (2018) [64]	Canada	To analyse the health knowledge, behaviours, and attitudes of truck drivers using a mixed-methods approach, to inform the design of more effective, targeted health promotion interventions.	Mixed Methods (forum analysis)	Online community posts (n = 1760)	Online health forum	Rasmussen’s Risk-Management Framework	Demonstrated health awareness but lacked deep knowledge; High self-blame, low awareness of systemic factors affecting health	High

* SHIFT—Safety and Health Involvement For Truck Drivers.

**Table 3:** Identified research gaps in the included studies.

Study	Key Research Gaps
Wipfli et al. (2019) [60]	Lack of assessment of long-term retention and post-intervention engagement; Absence of analysis isolating the relative contribution of specific digital components (e.g., mobile app versus coaching); and Limited explicit examination of intervention mechanisms using formal behavioural or technology acceptance frameworks.
Greenfield et al. (2016) [14]	Focus on perceptions and attitudes toward wearable technologies rather than observed use or engagement behaviour; Absence of behavioural outcome measures such as compliance or retention; Reliance on self-reported qualitative data without validation through usage metrics; and Lack of tested or proposed design strategies to address identified barriers.
Heaton et al. (2017) [61]	Examination of access to and interest in digital health tools without implementation or evaluation of perceived engagement; Absence of adherence, retention, or sustained usage outcomes; and Descriptive reporting of behavioural factors without theoretical modelling.
Levi-Bliech et al. (2019) [62]	Inclusion of fleet drivers without explicit differentiation of occupational road freight or long-haul trucking context; Limited focus on health-related behaviour change and sustained engagement; and Absence of analysis linking mobile app use to transport-specific working conditions.
Crizzle et al. (2022) [63]	Emphasis on mandated compliance outcomes rather than voluntary adoption or engagement processes; and Limited examination of user acceptance, motivation, or behavioural adaptation to the technology.
Versteeg et al. (2018) [64]	Reliance on secondary online forum data with limited representativeness of the wider truck driver population; Absence of evaluation of intervention effectiveness or structured e-health engagement outcomes; and Lack of translation from informal digital interaction to sustained, designed e-health interventions.

**Table 4:** Summary of synthesised findings according to the five predefined research questions.

Research Question	Summary of Synthesised Findings
RQ1: Engagement, Compliance, and Retention	**Engagement:** Driven by self-monitoring, real-time feedback, and peer support; stronger when tools were simple, adaptive, and routine-aligned; declined with complex or time-demanding systems ^(1)(2)(4)(5)(6)^ **Compliance:** Improved through motivational coaching, goal setting, and job-related incentives ^(1)(5)(6)^ ***Retention:*** Supported by perceived value, usability, and automation; weakened by high effort or manual input ^(2)(3)(5)^
RQ2: User Demographics, Preferences, and Needs	**Demographics:** Younger, more educated, and less experienced drivers used digital tools more frequently and with greater ease; older or less tech-savvy drivers reported usability challenges and frustration ^(3)(5)(6)^ **Preferences:** Consistently favoured simple, accessible, and job-compatible applications that required minimal setup and matched the realities of mobile work ^(1)(2)(6)^ **Needs:** Drivers expressed a desire for personalised, relevant, and flexible interventions (e.g., in-cab exercises, brief modules, and practical health guidance designed for long travel) ^(1)(2)(6)^
RQ3: Barriers and Challenges	**Occupational barriers:** Long shifts, irregular schedules, and limited rest made consistent participation difficult; fatigue and physical strain further hindered engagement ^(1)(2)(3)^ **Environmental constraints:** Limited access to healthy food, safe parking, and rest areas restricted drivers’ ability to follow interventions ^(1)(5)^ **Technological barriers:** Perceived complexity, login friction, and system errors disrupted use; rigid or time-locked systems conflicted with personal routines ^(4)(5)^ **Psychological and organisational factors:** Fear of employer surveillance, job loss, and judgement reduced trust; stigma and pre-existing health misinformation, particularly about mental health, limited willingness to participate openly (without stigma-related hiding) ^(2)(6)^
RQ3.1: Barriers and Challenges Variation Across Groups/Contexts	**Individual differences:** Age, digital literacy, and experience strongly influenced adoption; older or less tech-savvy drivers faced more adaptation difficulties, while younger and more educated drivers engaged more easily ^(3)(5)^ **Work context:** Drivers with irregular or high-variability routes experienced greater time pressure, fewer rest opportunities, and more usability challenges, whereas those with more stable routines encountered fewer of these barriers ^(2)(5)^ **Engagement patterns:** Differences in motivation and routine produced variable participation, with early engagement spikes and preferences for passive over active interaction ^(1)(4)^ **Perceptions of responsibility:** Some drivers attributed poor health outcomes to personal choices rather than systemic conditions, masking context-specific barriers ^(6)^
RQ4: Role of Technology Design	**Interface design:** Engagement improved with simple, intuitive, and visually appealing designs that supported easy navigation and mobile accessibility ^(1)(2)^ **Feedback and automation:** *Real-time feedback*, *automated alerts*, and *goal-tracking* effectively maintained motivation and supported behavioural self-regulation ^(1)(4)(5)^ **Gamification and personalisation:** Gamified elements such as anonymized team-based rankings, progress displays, and personalised goals encouraged interaction but were underutilised across studies ^(1)^ **Gaps in innovation:** Few studies addressed personalisation, gamification, or data security, revealing a lack of persuasive and user-centred design strategies in current occupational e-health tools ^(3)(6)^

^(1)^ Wipfli et al. (2019) [60]; ^(2)^ Greenfield et al. (2016) [14]; ^(3)^ Heaton et al. (2017) [61]; ^(4)^ Levi-Bliech et al. (2019) [62]; ^(5)^ Crizzle et al. (2022) [63]; ^(6)^ Versteeg et al. (2018) [64].

**Table 5:** Summary of alignment with study findings according to the six theoretical frameworks.

Theoretical Framework	Construct	Summary of Alignment with Study Findings
UTAUT2	Performance Expectancy	Perceived usefulness; improved health and safety outcomes; enhanced work performance ^(1)(2)(3)(4)(5)^
	Effort Expectancy	Ease of use; simple interfaces; low time demand; alignment with work routines ^(1)(2)(4)(5)^
	Facilitating Conditions	Access to digital tools; technical and organisational support; digital literacy ^(1)(3)(4)(5)^
	Social Influence	Peer encouragement; team participation; collective motivation ^(1)(2)^
	Habit	Routine integration; repeated voluntary interaction; sustained engagement patterns ^(3)^
MAVA	Trust in Automation	Reliability and consistency of automated systems; perceived fairness and predictability ^(2)(5)^
	Perceived Safety	Confidence that automation improves safety, reduces fatigue, and supports compliance ^(5)^
	Perceived Control	Desire for autonomy and flexibility; frustration with rigid or automated structures ^(5)^
	System Transparency	Understanding of how data is collected, shared, and monitored; privacy awareness ^(2)^
	Organisational Trust	Belief that employers will not misuse data; job security and ethical data handling ^(2)(5)^
SDT	Autonomy	Voluntary participation; goal setting; flexibility in use; reduced autonomy due to rigid system structures or surveillance ^(1)(2)(4)(5)(6)^
	Competence	Confidence in using digital tools; skill development via feedback, self-monitoring, and progress tracking ^(1)(2)(4)(5)(6)^
	Relatedness	Team support; peer competition; social encouragement; collective engagement in health behaviours ^(1)(2)(6)^
COM-B	Capability	Health literacy; digital competence; learning through self-monitoring and feedback ^(1)(2)(4)(5)(6)^
	Opportunity (Physical)	Access to technology; work conditions; available rest, time, and safe parking ^(1)(2)(3)(5)(6)^
	Opportunity (Social)	Peer encouragement; employer and organisational support; collaborative culture ^(1)(2)(6)^
	Motivation (Reflective)	Goal-setting; perceived usefulness; intention to maintain health; evaluation of personal progress ^(1)(2)(4)(5)(6)^
	Motivation (Automatic)	Habit formation through repeated app use; competition; gamified or incentive-based engagement ^(1)(4)(5)^
HBM	Perceived Susceptibility	Awareness of personal health risks (fatigue, chronic illness, stress) ^(2)(3)(6)^
	Perceived Severity	Fear of illness and preventive motivation ^(2)^
	Perceived Benefits	Belief that tools improve safety, compliance, and overall well-being ^(3)(5)^
	Perceived Barriers	Privacy concerns, job insecurity, system inflexibility, misinformation, stigma ^(2)(5)(6)^
	Self-Efficacy	Confidence in ability to use technology and maintain healthy behaviour ^(1)(4)(5)^
	Cues to Action	Employer mandates, certifications, and reminders prompting engagement ^(1)(5)^
PSD	Primary Task Support	Goal tracking; behavioural feedback; progress visualisation; simplicity of use ^(1)(2)(4)^
	Dialogue Support	Real-time feedback; automated prompts; motivational reinforcement ^(1)(4)(5)^
	Social Support	Peer encouragement; team competition; community-based interaction ^(1)(6)^

^(1)^ Wipfli et al. (2019) [60]; ^(2)^ Greenfield et al. (2016) [14]; ^(3)^ Heaton et al. (2017) [61]; ^(4)^ Levi-Bliech et al. (2019) [62]; ^(5)^ Crizzle et al. (2022) [63]; ^(6)^ Versteeg et al. (2018) [64].

**Table 6:** Cross-study summary of determinants on compliance, retention, and long-term engagement of truck drivers to e-health and mobile applications.

Level	Determinants	Description	(1)	(2)	(3)	(4)	(5)	(6)
**BEHAVIOURAL** **(Individual)**	**Self-monitoring &** **Behaviour Tracking**	Use of tools to log, monitor, or receive feedback on behaviours (e.g., weight, sleep, driving)	✔			✔	✔	
	**Social Motivation &** **Incentives**	Group-based competition, rankings, or rewards (e.g., certification, gift cards)	✔					✔
	**Health Coaching &** **Motivational Support**	Use of goal-setting, interviews, or guided (external) support for behaviour change	✔					✔
	**Personalisation &** **Goal Customisation**	The ability for users to set personal goals or receive tailored content based on preferences or behaviour.	✔	✔		✔		
	**Engagement Pattern over Time**	Whether engagement is sustained, declines quickly, or varies by timing/context	*			*		
	**Digital Literacy &** **Demographics**	Influence of age, education, or experience on ability or willingness to adopt tech		✔	✔		✔	
	**Perceived Usefulness or Value**	Drivers’ belief that the tool helps them (or does not) manage health or safety	✔		✔	✔	✔	✔
**TECHNOLOGICAL** **(System)**	**Feedback & Reminders**	Prompts, progress updates, or cues that help users stay on track with tasks or health goals.	✔			✔	✔	✔
	**Gamification/Progress** **Visualisation**	Use of visual tools (e.g., rankings, badges, graphs) to reinforce progress and motivate engagement.	✔			✔		
	**Real-Time Feedback &** **Responsiveness**	Immediate system responses or prompts based on user input or behaviour during real-time use.	✔			✔	✔	
	**Technological Simplicity & Usability**	Perception that the tool is easy or hard to use; the effort required to learn it		✔		✔	*	*
	**Technology Access &** **Infrastructure**	Access to devices and health apps, Wi-Fi, app logins, or on-road digital tools	✔		✔	*		*
**CONTEXTUAL (Work and** **Environment)**	**Work schedule & Fatigue**	Long shifts, irregular hours, and limited sleep affect the ability to comply or engage	*	✔			✔	✔
	**Relevance to Driver Context**	Whether the tool fits trucker routines, cab environments, or mobile life	✔	✔			✔	✔
	**Privacy & Employment** **Concerns**	Fears about employer surveillance, data misuse, or job risks		✔			✔	

✔ = present; * = conditionally influential.

## Data Availability

No new data were created or analysed in this study.

## Supplementary Materials

The following supporting information can be downloaded at: https://www.mdpi.com/article/10.3390/healthcare14030340/s1; The supplementary materials contain the PRISMA checklist and a detailed framework of the methodology applied (e.g., search strategy, eligibility criteria, data categorisation framework, screening phases, quality assessment, etc.). References [16,17,45,46,47,48,49,50,51,52,53,54,55,56,57,58,59,65,91,94,95,96] cited in Supplementary file.

## Abbreviations

The following abbreviations are used in this manuscript:

AI	Artificial Intelligence
CASP	Critical Appraisal Skills Programme
COM-B	Capability, Opportunity, Motivation—Behaviour model
ELD	Electronic Logging Device
HBM	Health Belief Model
IRU	International Road Transport Union
IoT	Internet of Things
MAVA	Multi-Level Model on Automated Vehicle Acceptance
MMAT	Mixed Methods Appraisal Tool
PRISMA	Preferred Reporting Items for Systematic Reviews and Meta-Analyses
PSD	Persuasive Systems Design
RQ1-RQ4	Research Questions 1 to 4
SDT	Self-Determination Theory
SHIFT	Safety and Health Involvement For Truckers
TRID	Transport Research International Documentation
UTAUT2	Extended Unified Theory of Acceptance and Use of Technology

## Appendix A

**Table A1:** Definitions of theoretical constructs.

Framework	Constructs	Definition
UTAUT2	Performance Expectancy	The belief that using the technology will improve task performance or outcomes.
	Effort Expectancy	The degree to which the system is perceived as easy to understand, learn, or use.
	Social Influence	The extent to which important others believe the user should adopt the technology.
	Facilitating Conditions	The availability of resources, support, and infrastructure needed to use the system.
	Hedonic Motivation	The enjoyment or pleasure derived from using the technology.
	Price Value	The perceived trade-off between benefits and any costs associated with using the technology.
	Habit	The extent to which behaviour becomes automatic due to repeated use.
MAVA	Individual Traits	Personal characteristics that influence how a user perceives and accepts technology.
	Demographics	Background variables (e.g., age, education) shaping technology attitudes.
	Personality	Stable traits influencing confidence, openness, or comfort with technology.
	Travel Behaviour	Driving routines and experiences affecting acceptance of technologies.
	Exposure	Previous experience with similar systems or technologies.
	Domain-Specific Factors	Contextual elements tied to the driving environment or task demands.
	Symbolic-Affective Factors	Emotional or symbolic meanings attached to the technology.
	Moral-Normative Factors	Ethical beliefs or social norms influencing acceptance.
SDT	Autonomy	Feeling in control of one’s actions and able to make choices freely.
	Competence	Feeling capable, effective, and confident in using the technology.
	Relatedness	Feeling connected and supported by others while engaging with the technology.
COM-B	Physical Capability	Physical skills or abilities required to perform the behaviour.
	Psychological Capability	Knowledge and mental skills needed to perform or understand the behaviour.
	Physical Opportunity	Environmental or physical conditions that enable the behaviour.
	Social Opportunity	Social contexts and interpersonal influences that support the behaviour.
	Automatic Motivation	Habitual, emotional, or impulse-driven processes influencing behaviour.
	Reflective Motivation	Conscious intentions, goals, and evaluations that guide behaviour.
HBM	Perceived Susceptibility	Belief in the likelihood of experiencing a health issue.
	Perceived Severity	Belief in the seriousness of the condition and its consequences.
	Perceived Benefits	Belief that performing the behaviour will reduce risks or improve outcomes.
	Perceived Barriers	Beliefs about obstacles that may prevent the desired behaviour.
	Cues to Action	Triggers that prompt behaviour, such as reminders or recommendations.
	Self-Efficacy	Confidence in one’s ability to perform the behaviour successfully.
PSD	Primary Task Support	Features that help users accomplish core tasks (e.g., tailoring, self-monitoring).
	Dialogue Support	Interactive features such as praise, reminders, rewards, or feedback.
	System Credibility Support	Features enhancing the system’s trustworthiness or professionalism.
	Social Support	Features that enable social interaction, comparison, or cooperation.

## Appendix B

**Table A2:** Study exclusion categories and corresponding codes.

Code	Reason for Exclusion	Applies at Phase
E0	Not eligible based on basic criteria (e.g., not peer-reviewed, language not in English, not published, conference abstract, retracted)	Pre-Screening
E1	The population is not truck drivers (e.g., young drivers, old drivers, regular car drivers)	Title, Abstract, Full-Text
E2	The study focuses on other transport sectors (e.g., aviation, maritime) or not the transportation sector; the target population is not clear.	Title, Abstract
E3	No digital health intervention	Title, Abstract, Full-Text
E4	Not focused on compliance, adoption, engagement, or usage of digital tools	Abstract, Full-Text
E5	Outcomes not relevant (e.g., unrelated health aspects or hardware-only monitoring)	Abstract, Full-Text
E6	Insufficient methodological detail/No access to full text	Full-Text
E7	Wrong publication type (e.g., editorial, comment, protocol, letter)	Full-Text
E8	Not a research study (e.g., introduction to Special Issue, commentary, opinion)	Full-Text
E9	Duplicate content is still detected manually (e.g., duplicate entry or dual publication)	Full-Text
