# Supplementary material for: Increasing Truck Drivers’ Compliance, Retention, and Long-Term Engagement with e-Health & Mobile Applications: A PRISMA Systematic Review
Source: Healthcare (Basel). 2026 Jan 29;14(3):340. doi: 10.3390/healthcare14030340 (PMC12897317; doi:10.3390/healthcare14030340)
Supplement: Supplementary file 1 [file healthcare-14-00340-s001.zip › healthcare-4054478-supplementary.pdf]

METHODOLOGY

1. Introduction

A protocol was developed to conduct a study with the highest possible methodological rigor and to minimize the risk of bias. The preparatory work for this protocol was carried out by three researchers. One researcher was responsible for the operationalization and execution of the protocol, while the other two researchers provided oversight. Although the screening and analysis were conducted by a single primary researcher, this approach has been employed in previous studies (e.g., South et al., 2019; Waffenschmidt et al., 2019; Wang et al., 2023). Regular consultations were held to discuss progress and to ensure adherence to the protocol. Whenever questions arose during the screening and analysis of studies by the primary researcher, these were discussed in detail with the other two researchers to reach consensus.

For this study, the structure and content of the review followed the PRISMA 2020 Item Checklist (Page et al., 2021a; Page et al., 2021b) to ensure methodological consistency and transparency.

This study' methodological steps for study selection followed a structured 10-step process aligned with the PRISMA protocol, as shown in the figure below.

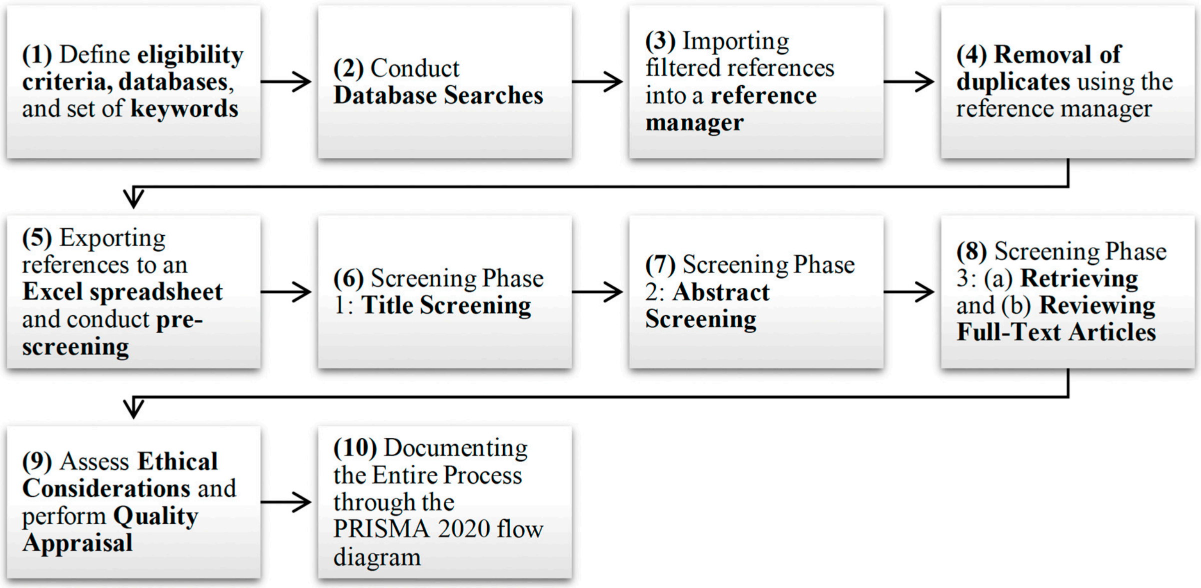

Figure S1: Structured 10-step study selection process based on the PRISMA 2020 protocol (Own elaboration)

The figure illustrates the sequential process used to guide study selection in this review, beginning with establishing eligibility criteria and progressing through database searches, reference management, duplicate removal, and multi-phase screening. Ethical assessment and methodological quality appraisal were incorporated prior to final inclusion decisions. The process concludes with the formal documentation of results following the PRISMA 2020 standards, ensuring transparency and replicability.

As part of the study selection process, all search results were exported from their respective databases and imported into Zotero, an open-source reference management tool. Zotero was used to organise citations, store full-text documents, and identify and remove duplicate records prior to the screening phases, corresponding to Steps 3 and 4 of the 10-step structured process outlined in the previous section. This method aligns with the deduplication process recommended under the PRISMA 2020 protocol.

Following deduplication, Steps 5 to 9 of the structured process, involving title screening, abstract screening, full-text screening, ethical assessment, and quality appraisal, were conducted using a customised Excel spreadsheet. The transition from Zotero to Excel ensured a transparent and traceable workflow throughout the systematic review process.

2. Search Strategy

A structured search strategy was strategically developed to identify, screen, and select relevant studies for inclusion in this review, which is a process in accordance with the PRISMA 2020 protocol. The search process was based on (1) clearly defined eligibility criteria, (2) multiple information sources, and the application of (3) comprehensive keywords and search terms. By combining these three elements, the search strategy ensured the retrieval of high-quality, relevant literature aligned with the research objectives while maintaining transparency and reproducibility throughout the review process. Each element is further discussed in the succeeding sections.

2.1 Eligibility Criteria

Specific eligibility criteria were established to determine the inclusion and exclusion of studies and ensure a focused and high-quality review. These criteria were designed to refine the search results and ensure that only relevant, peer-reviewed literature addressing digital interventions for trucks drivers in the transportation sector was considered.
Studies were included if they met the following criteria:

- They focused on digital health interventions (e-health, mHealth, telemedicine) for truck drivers within the transportation sector.
- They were peer-reviewed journal articles to ensure the credibility and reliability of the findings.
- They were published in English to maintain consistency in data analysis and avoid language barriers.
- Studies were excluded if they:
- Focused on digital interventions for non-occupational drivers or general transportation users rather than truck drivers.
- Did not involve digital health technologies or lacked a connection to compliance and user adoption in the transportation sector.
- Were conference papers, books, book chapters, editorials, or retracted articles, as these sources do not always undergo rigorous peer review.
- They were not published in English.

2.2 Information Sources

Multiple reputable databases were selected as information sources for this review, given their recognised suitability for systematic literature searches (Gusenbauer & Haddaway, 2020). Their relevance to the research topic guided the choice of databases, their coverage of peer-reviewed studies, and their strengths in capturing multidisciplinary work across health, transportation, and occupational research. Advanced search strategies were developed for each database to ensure consistency in search terms while tailoring the syntax to match the unique search functionalities of each platform. The following databases were used:

1. PubMed

PubMed was selected for its extensive indexing of biomedical and health-related literature, including studies focused on digital health interventions (Ossom Williamson & Minter, 2019). It offers a robust, controlled vocabulary system (MeSH terms) and reliable peer-reviewed content. Advanced searches were conducted using a combination of MeSH terms and keywords, applying the [All Fields] tag to maximise coverage of both indexed and newly published articles (Richter & Austin, 2012).

2. Scopus

Scopus, acknowledged as the largest abstract and citation database for scientific literature, was included for its broad coverage of scientific, technical, and social science literature, providing access to peer-reviewed journals, conference proceedings, and books (Meho & Yang, 2007; Schotten et al., 2017). The advanced search strategy employed in Scopus utilised Boolean operators to combine key concepts. It targeted all searchable fields rather than restricting the search to specific sections such as titles, abstracts, or keywords. This approach ensured a comprehensive retrieval of studies relevant to digital health interventions within the transportation sector.

3. Web of Science (WoS)

Web of Science, the oldest citation index for the sciences, was chosen due to its multidisciplinary indexing of high-quality peer-reviewed studies across health sciences, social sciences, and engineering (Birkle et al., 2020). The advanced search function used topic searches (TS=) to locate relevant studies based on titles, abstracts, author keywords, and Keywords Plus. The platform's filtering options for document type, language, and research area ensured alignment with the inclusion criteria.

4. Transport Research International Documentation (TRID)

TRID was selected for its specialised focus on transportation research. According to the TRID website, it is a unified database that merges records from the Transportation Research Board's (TRB) Transportation Research Information Services (TRIS) Database and the International Transport Research Documentation (ITRD) Database maintained by the OECD's Joint Transport Research Centre (TRB, n.d.). Given the review's focus on occupational drivers within the transportation sector, TRID's subject indexing allowed precise targeting of technical reports and studies relevant to freight and road transport. Similar to the other databases, searches were structured using title keywords and subject terms to capture the most relevant studies.

2.3 Keywords and Search Terms

As previously stated, an advanced search strategy was employed across multiple databases to conduct a comprehensive literature search. A combination of keywords and Boolean operators (AND, OR) was utilised to construct and refine the search queries, ensuring a systematic retrieval of relevant studies. The application of filters, such as language and document type restrictions, was aligned with the eligibility criteria.

Because the search aimed to identify relevant studies examining compliance, retention, and engagement with digital health technologies within the transportation sector, specifically among truck drivers, the search strategy was structured around the following key concepts: compliance, retention, user engagement, e-health, transportation sector, and truck drivers. To ensure comprehensive coverage of the literature, the initial development of the search string was supported by the use of an AI tool (ChatGPT), which helped generate a broad list of potential keywords and Boolean combinations.

The resulting search string was subsequently refined and assessed by the author to ensure relevance, accuracy, and alignment with the study's objectives. The final search string was as follows:

(compliance OR conformity OR observance OR commitment OR retention OR continuation OR engagement OR participation OR involvement OR motivation OR user adoption)
AND
(e-health OR digital health OR telemedicine OR mHealth OR online health OR virtual health OR electronic health)
AND
(transportation sector OR transportation OR transport sector OR transport OR mobility OR transit OR traffic systems OR traffic)
AND
((truck OR heavy vehicle OR freight OR commercial OR long-haul OR professional OR logistics OR delivery) AND (driver OR operator*))*

All databases used Boolean operators, phrase searching using quotation marks, and field-specific searches targeting titles, abstracts, or subject areas. Additionally, all databases offer filtering options, such as publication date, document type, and language, to refine search results. However, there are still minor differences in how each database executes advanced searches.

3. Data Extraction and Analysis

This section outlines the procedures used to manage, screen, evaluate, and interpret the literature included in this review. It begins with the development of a data categorisation framework, detailing how studies were organised and what variables were extracted. This is followed by a description of the multi-phase screening process used to determine study eligibility. Sequentially, ethical considerations related to the included studies were assessed. Finally, the quality assessment approach, which evaluates the methodological robustness of each study to support transparent and meaningful studies, was done. These components form the foundation for the subsequent interpretation and analysis of findings.

3.1 Data Categorisation Framework

A data categorisation framework was developed as part of the review process to ensure a structured and transparent approach to managing the filtered literature from chosen databases. After removing duplicates in Zotero, the remaining studies were exported to an Excel spreadsheet, which served as the main tool for managing the screening process, recording inclusion and exclusion decisions, and organizing data for analysis. The use of Excel allowed for systematic monitoring of each study across the different phases of screening: (1) title screening, (2) abstract screening, and (3) full-text screening, ensuring that decisions were consistently applied at every stage. It also includes the processes of ethical approval, quality assessment, and final data extraction.

3.1.1 Categorisation Criteria

The selected studies were organised within the Excel spreadsheet according to a structured set of categorisation criteria. These criteria were developed to capture essential information at different stages of the review systematically and to maintain consistency throughout the data extraction process. To reflect the stages of study processing, the variables were divided into two groups: (1) variables automatically extracted by Zotero during the initial screening phase and (2) variables manually extracted during full-text review.

Table S1 presents the pre-determined variables automatically extracted from Zotero after initial database searches and screening. These Zotero-extracted variables represent common bibliographic elements typically embedded in citation metadata and are automatically captured by most reference management systems. As such, they do not follow a specific methodological framework or theoretical basis but rather reflect standard fields used in citation indexing. Zotero offers a wide range of metadata fields, but the researcher selected only those considered most relevant to the title and abstract screening process and data synstudy. This selection was guided by professional judgment and the practical needs of the review, such as tracking source origin, verifying eligibility criteria (i.e., language and publication type), and organizing records consistently across databases.

Table S1: Pre-determined variables extracted from Zotero after initial database searches and screening (Own elaboration)

**Field/Column**	**Description of Importance**
Study ID	A unique number/code assigned to each study for tracking & referencing
Title of the Study	Useful for identification and quick review
Item Type	Determines the reference type (e.g., journal, etc.
Year of Publication	Assess the recency and relevance of the study
Author(s)	Helps identify the study and cite it correctly
Publication Title	Identifies where the reference is published
Abstract Note	Useful for abstract screening
Language	Helpful in verifying if the study meets the language criteria
Database Source	To track where the study was taken

Table S2 outlines the variables extracted during the full-text review phase, which took place only after studies passed all prior screening steps (i.e., title and abstract screening). These studies were considered eligible for full-text review and were therefore subjected to more detailed data extraction. The selected variables reflect key study characteristics that

were critical for interpretation, appraisal, and synstudy, such as study design, population, country, intervention type, outcomes measured, and theoretical framework.

The choice of these variables was informed by established practices in systematic review methodologies (i.e., Hoque et al., 2020; Indra et al., 2024; Ng et al., 2015), where similar parameters are often used to organize and compare complex evidence across diverse studies. Each field was chosen based on its relevance to understanding the context and applicability of the included studies in relation to the research objectives of this review.

Table S2: Extracted variables based on full-text review for data extraction and analysis (Own elaboration based on Hoque et al., 2020; Indra et al., 2024; Ng et al., 2015)

**Field/Column**	**Description of Importance**
Study Design	To identify the appropriate appraisal tool, for assessing the quality of the study and level of evidence for additional transparency
Country	To identify the study's geographic context, relevant for understanding regional differences
Population	Description of participants
Sample Size	Helps assess the strength and validity of findings
Intervention Type	Type of digital health intervention applied in the study
Comparison Group	If any; Relevant for evaluating outcomes
Study Duration	Timeframe of the study; helps assess sustainability and exposure
Setting	Workplace, on-road, clinical, remote, etc.; context for how the intervention was implemented
Data Collection Methods	Helps assess validity
Digital Platform/Tool Used	Names/Types of tools used
Theoretical Framework	Any behavioural or health theory used - Useful for conceptual mapping
Peer-Reviewed	Final checking to ensure that the inclusion criteria are met
Funding Source	Reveals potential bias or conflict of interest
Limitations Noted	To acknowledge weaknesses in the study; helps in quality appraisal
Ethical Consideration	To assess if a study was carried out ethically
Key Findings	Summary of main results; forms the basis for synthesis
Outcomes Measured	Primary outcomes (e.g., engagement, compliance, retention, health indicators)

3.1.2 Data Extraction Tables

The earlier discussed parameters served as the foundation for constructing the data extraction tables. Each study was assigned a single row, while each column reflected a specific parameter outlined in the categorisation criteria. The tables were designed to facilitate the consistent tracking of study characteristics, screening outcomes, eligibility decisions, ethical assessments, and quality appraisal results and to organise the necessary information to fill out the PRISMA Flow Diagram. Excel was selected as the data management tool due to its flexibility in sorting, filtering, and updating information throughout the screening and analysis phases (Pernsley, 2016). The data extraction table was progressively updated as the review advanced through its stages, ensuring transparency and traceability in decision-making.

A snapshot of the Excel file data extraction table tabs is presented below to illustrate the organisation of information across studies and how the author conducted the systematic review.

Summary	Deduplicated records_screening	Full-Text Review - Details	Legend

Figure S2: Tabs of the customised Excel file used for study screening and data extraction (Own work)

As seen in Figure S2, the data extraction and screening processes were managed through a customised Excel workbook containing multiple organised sheets, each serving a distinct function:

1. Summary

This sheet provides an overview of the screening and data extraction progress. It compiles key figures needed for the PRISMA 2020 flow diagram, such as the number of records identified, screened, excluded, and included. See Figure S3 for the snapshot of the content of this sheet.

					Based on Eligi	
	**DATABASES**	**No. of Filtered Literature (Initial)**	Removed Duplicates (within Multiple Databases)	***Pre-Screening***	**(1) For Title Screening**	**(2) For Abstract Screening**
1	**PubMed**	99	7	647	644	26
2	**Scopus**	505				
3	**Web of Sciences**	42				
4	**TRID**	8				
	**Total**	**654**	***Removed Records =***	***3***	***618***	***9***

Based on Eligibility Criteria				*Based on Quality*		
**(2) For Abstract Screening**	**(3a) Reports Sought for Retrieval**	**(3b) For Full-Text Screening**	**Studies included in Review for Data Analysis**	**(1) CASP for Quantitative Studies**	**(2) CASP Qualitative Checklist**	**(3) MMAT**
26	17	16	**6**	3	1	2
9	***1***	***10***				

Figure S3: Summary tab: Overview of screening and data extraction progress (Own work)

2. Deduplicated records_screening

This sheet contains all the extracted bibliographic information imported from Zotero. It served as the main working file for title screening, abstract screening, retrieval availability checks, and full-text screening.

3. Full-Text Review – Details

Studies that passed the title screening, abstract screening, and retrieval availability were transferred to this sheet. It was used to extract detailed study characteristics from the entire article, including the ethical considerations (see section 3.3), to assess the full text according to the eligibility criteria, and to perform quality assessments (see section 3.4) using appropriate critical appraisal tools. During the full-text extraction, an AI tool (ChatGPT) was also used to assist in summarizing and organizing key study details; however, all critical decisions (i.e., assessing eligibility, assigning quality ratings, and

interpreting content) were made by the researcher.

4. Legend

This sheet provides a quick reference guide listing all exclusion reasons (E0–E9) (see Table S3), categories for ethical approval (see Table S4), and the corresponding appraisal tools (see Table S5) selected based on the type of study design.

3.2 Screening Phases

The screening process was conducted in three sequential stages: title screening, abstract screening, and full-text screening (Page et al., 2021a; Page et al., 2021b). Each stage progressively refined the pool of identified studies to ensure that only those meeting the eligibility criteria advanced to the next phase. Prior to the title screening, the researcher conducted a pre-screening to double-check whether the studies extracted from Zotero meet the basic criteria, such as the language and document type (if peer-reviewed article).

During title screening, studies were excluded based on clear mismatches in population focus, sector relevance, or intervention type. Abstract screening allowed for a more detailed evaluation of study relevance based on intervention focus, target outcomes, and study population. Full-text screening provided the most comprehensive assessment, confirming eligibility against all predefined criteria, including methodological quality assessment.

A set of exclusion codes was applied consistently across all screening stages to document the specific reasons for study removal. Screening decisions and corresponding exclusion codes were recorded systematically in the data extraction table.

Table S3: Reasons for study exclusion across screening phases and their corresponding codes (Own elaboration)

**Code**	**Reason for Exclusion**	**Applies at Phase**
E0	Not eligible based on basic criteria (e.g., not peer-reviewed, language not in English, not published, conference abstract, retracted)	Pre-Screening
E1	The population is not truck drivers (e.g., young drivers, old drivers, regular car drivers)	Title, Abstract, Full-Text
E2	The study focuses on other transport sectors (e.g., aviation, maritime) or not the transportation sector; the target population is not clear.	Title, Abstract
E3	No digital health intervention	Title, Abstract, Full-Text
E4	Not focused on compliance, adoption, engagement, or usage of digital tools	Abstract, Full-Text
E5	Outcomes not relevant (e.g., unrelated health aspects or hardware-only monitoring)	Abstract, Full-Text
E6	Insufficient methodological detail / No access to full text	Full-Text
E7	Wrong publication type (e.g., editorial, comment, protocol, letter)	Full-Text
E8	Not a research study (e.g., introduction to special issue, commentary, opinion)	Full-Text
E9	Duplicate content is still detected manually (e.g., duplicate entry or dual publication)	Full-Text

3.3 Ethical Considerations

For additional information, the ethical approval status was recorded for all included studies that underwent full-text screening. The purpose of documenting the ethics approval was to assess the ethical integrity of the studies and to provide a clear account of research practices involving human participants, even though it was not used as a basis for exclusion. Because ethical approval was not part of the original inclusion and exclusion criteria, studies were not excluded solely based on the absence of an ethics statement. This means studies without explicit proof of ethical approval were still eligible for inclusion in the review.

Documenting the ethics approval status served two key functions. First, it enabled a more transparent and responsible evaluation of the quality and credibility of included studies, particularly when assessing the handling of human data. Second, it allowed the researcher to reflect on and report potential ethical gaps across the body of literature. This contributes to the broader discussion about research standards in the field and highlights areas where ethical reporting needs to be improved in future studies. In this way, even though ethics approval did not directly influence inclusion, recording it added value by strengthening the review's integrity and offering insights into ethical practices in digital health and transport-related research.

Table S4 outlines the classification categories for recording the ethics approval status across the reviewed studies.

Table S4: Ethics approval categories for included studies in full-text screening (Own elaboration)

**Ethics Approval**	**Notes**
Yes	Approved by an Institutional Review Board (IRB) or equivalent ethics committee
No	No mention of ethics approval
Waived	Ethics approval was waived due to the use of anonymised or publicly available data
Unclear	Ethics approval status is not specified in the full text.

3.4 Quality Assessment of Selected Studies

Each included study was critically appraised using appraisal tools appropriate to its design to assess its methodological soundness. This section begins by identifying the appraisal tools used across different study types, followed by an explanation of the scoring system applied to interpret appraisal results. The final subsection describes how these quality ratings informed inclusion decisions and guided the interpretation of evidence in the synstudy phase.

3.4.1 Appraisal Tools

Critical appraisal tools are structured checklists or frameworks designed to assess the methodological quality, credibility, and risk of bias in research studies. They help reviewers systematically evaluate the reliability of study findings by focusing on aspects such as research design, data collection, and the measures taken to reduce potential biases (Katrak et al., 2004; Munn et al., 2015).

Depending on the study design, a specific appraisal tool was applied. These tools were selected due to their widespread use in evidence synstudy, adaptability across diverse research designs, and ability to provide a structured and transparent assessment of study quality (Hong et al., 2019; Long et al., 2020).

The CASP (Critical Appraisal Skills Programme) checklists were selected as the appraisal tool for quantitative and qualitative studies to maintain consistency and standardisation across the review process. Using CASP ensured that all necessary checklists came from a single, accessible source (i.e., (CASP, n.d.)), removing the need to search for multiple appraisal tools. CASP was also chosen because it is easy to use, clearly structured, widely available, and accepted in evidence-based research for evaluating the methodological quality of different study designs (Long et al., 2020).

However, in the case of mixed-methods studies, the MMAT (Mixed Methods Appraisal Tool) was applied due to its integrated approach in assessing both qualitative and quantitative components (Hong et al., n.d.; Hong et al., 2019). The assignment of appraisal tools according to study type is summarized in Table S5.

Table S5: Appraisal tools applied according to study design (Own elaboration)

Study Design	Examples	Appraisal Tool
Quantitative	Randomised controlled trials (RCTs), interventional trials	CASP
Qualitative	Interview-based studies, focus group research, thematic analysis studies	CASP
Mixed Methods	Combined quantitative and qualitative studies	MMAT

Specific assessment criteria varied depending on the selected appraisal tool, as each instrument targets different methodological fields relevant to the study design under evaluation. In line with this, this study will not discuss the assessment criteria in detail.

3.4.2 Scoring System

Each included study was assessed across relevant domains, such as clarity of research aims, appropriateness of methodology, recruitment strategy, data collection, ethical considerations, and robustness of findings. Studies were rated as high, moderate, or low quality based on the degree to which they met the appraisal criteria.

The purpose of the ratings was to allow clearer categorization of the evidence base and to help contextualize the credibility and weight of individual study findings within the broader review, making the synstudy and interpretation more manageable and meaningful than relying on numerical scores alone.

Since each appraisal tool is designed with different assessment criteria and structures, their scoring systems follow slightly different approaches. The CASP checklists consist of approximately 10–13 questions, depending on the study type. On the other hand, the MMAT includes five (5) core criteria. For simplicity, a standard scoring system was applied based on the number of "Yes" responses.

Tables S6 and S7 show the scoring approach that was used in CASP and MMAT, respectively.

Table S6: CASP scoring system (Own elaboration)

**Quality Level**	**Criteria**
High	8–10 (or 10-13) "Yes" answers, no serious concerns
Moderate	5–7 (or 6-9) "Yes" answers, some limitations but still usable
Low	Fewer than 5 (or 6) "Yes" answers, significant concerns about rigour or bias

Table S7: MMAT scoring system (Own elaboration)

**Quality Level**	**Criteria**
High	Meets all 5 criteria (5/5 Yes)
Moderate	Meets 3–4 criteria
Low	Meets 2 or fewer criteria

3.4.3 Inclusion Decision Based on Quality

Similar to the treatment of ethical approval status, quality assessment ratings were documented for all included studies but were not used as exclusion criteria during study selection. This approach was consistent with the goal of ensuring comprehensive coverage of the available evidence.

Rather than serving as a filtering mechanism, the quality assessment contributed to the review by informing the level of confidence placed on individual studies during synthesis. High- and moderate-quality studies were interpreted in relation to their methodological strengths, with noted limitations acknowledged in the analysis. This strategy supported transparency while allowing meaningful differentiation in the weight assigned to each study's findings.

Documenting study quality also added value by highlighting patterns of methodological strength across the included literature. This not only enabled a more nuanced synthesis but also helped identify areas where future research design and reporting could be further strengthened.

Table S8 summarizes how the quality ratings were used to guide the interpretation of studies during the synthesis phase.

Table S8: Study inclusion and interpretation based on quality assessment (Own elaboration)

**Quality Level**	**Scenario**	**Inclusion Decision & Interpretation**
High	High-quality studies with no major concerns; Meets nearly all quality criteria; robust methodology and reporting.	Included – Findings considered reliable with no major concerns
Moderate	It meets many criteria with some minor limitations in methodology or reporting; it still provides valuable insights.	Included – Findings considered valid, but note minor limitations
Low	Notably, several quality criteria were missing; with significant limitations or potential bias.	Included – Interpret findings with caution due to limitations

4. PRISMA 2020 Checklist

Table S9: PRISMA 2020 Checklist

Section and Topic	Item #	Checklist item	Location where item is reported
**TITLE**			
Title	1	Identify the report as a systematic review.	**Title,** *Page 1*
**ABSTRACT**			
Abstract	2	See the PRISMA 2020 for Abstracts checklist.	**Abstract,** *Page 1*
**INTRODUCTION**			
Rationale	3	Describe the rationale for the review in the context of existing knowledge.	**Introduction** (Section 1)**,** *Pages 1 & 2*
Objectives	4	Provide an explicit statement of the objective(s) or question(s) the review addresses.	**Study Objectives** (Section 2), *Page 3*
**METHODS**			
Eligibility criteria	5	Specify the inclusion and exclusion criteria for the review and how studies were grouped for the syntheses.	**Methodology** (Section 3), *Page 5*
Information sources	6	Specify all databases, registers, websites, organisations, reference lists and other sources searched or consulted to identify studies. Specify the date when each source was last searched or consulted.	**Methodology** (Section 3), *Pages 4 & 5*
Search strategy	7	Present the full search strategies for all databases, registers and websites, including any filters and limits used.	**Methodology** (Section 3), *Pages 4 & 5*
Selection process	8	Specify the methods used to decide whether a study met the inclusion criteria of the review, including how many reviewers screened each record and each report retrieved, whether they worked independently, and if applicable, details of automation tools used in the process.	**Methodology** (Section 3), *Pages 5 & 6*
Data collection process	9	Specify the methods used to collect data from reports, including how many reviewers collected data from each report, whether they worked independently, any processes for obtaining or confirming data from study investigators, and if applicable, details of automation tools used in the process.	**Methodology** (Section 3), *Pages 5 & 6*
Data items	10a	List and define all outcomes for which data were sought. Specify whether all results that were compatible with each outcome domain in each study were sought (e.g. for all measures, time points, analyses), and if not, the methods used to decide which results to collect.	**Methodology** (Section 3), *Page 5*
	10b	List and define all other variables for which data were sought (e.g. participant and intervention characteristics, funding sources). Describe any assumptions made about any missing or unclear information.	**Methodology** (Section 3), *Pages 5 & 6*
Study risk of bias assessment	11	Specify the methods used to assess risk of bias in the included studies, including details of the tool(s) used, how many reviewers assessed each study and whether they worked independently, and if applicable, details of automation tools used in the process.	**Methodology** (Section 3), *Page 5*
Effect measures	12	Specify for each outcome the effect measure(s) (e.g. risk ratio, mean difference) used in the synthesis or presentation of results.	*Not applicable*

Section and Topic	Item #	Checklist item	Location where item is reported
Synthesis methods	13a	Describe the processes used to decide which studies were eligible for each synthesis (e.g. tabulating the study intervention characteristics and comparing against the planned groups for each synthesis (item #5)).	**Methodology** (Section 3), *Page 5*
	13b	Describe any methods required to prepare the data for presentation or synthesis, such as handling of missing summary statistics, or data conversions.	*Not applicable*
	13c	Describe any methods used to tabulate or visually display results of individual studies and syntheses.	**Methodology** (Section 3), *Page 5;* **Results** *– Tables 2 – 5 ; Figures 1 & 2*
	13d	Describe any methods used to synthesize results and provide a rationale for the choice(s). If meta-analysis was performed, describe the model(s), method(s) to identify the presence and extent of statistical heterogeneity, and software package(s) used.	**Methodology** (Section 3), *Page 5;*<br>**Results** (Section 4.3), *Page 15*
	13e	Describe any methods used to explore possible causes of heterogeneity among study results (e.g. subgroup analysis, meta-regression).	*Not applicable*
	13f	Describe any sensitivity analyses conducted to assess robustness of the synthesized results.	*Not applicable*
Reporting bias assessment	14	Describe any methods used to assess risk of bias due to missing results in a synthesis (arising from reporting biases).	**Methodology** (Section 3), *Pages 5 & 6;* **Limitations** (Section 7), *Page 21*
Certainty assessment	15	Describe any methods used to assess certainty (or confidence) in the body of evidence for an outcome.	**Methodology** (Section 3), *Pages 5 & 6;* ***Certainty and Strength of Evidence*** (Section 6), *Pages 20-21*
**RESULTS**			
Study selection	16a	Describe the results of the search and selection process, from the number of records identified in the search to the number of studies included in the review, ideally using a flow diagram.	**Results** (Section 4.1), *Page 6 & 7; Figure 1*
	16b	Cite studies that might appear to meet the inclusion criteria, but which were excluded, and explain why they were excluded.	**Results** (Section 4.1), *Page 6*
Study characteristics	17	Cite each included study and present its characteristics.	**Results** (Section 4.2), *Pages 7 – 8; Table 2*
Risk of bias in studies	18	Present assessments of risk of bias for each included study.	**Discussion** (Section 5.1), *Pages 14 – 15;* **Limitations** (Section 7); *Page 21.*
Results of individual studies	19	For all outcomes, present, for each study: (a) summary statistics for each group (where appropriate) and (b) an effect estimate and its precision (e.g. confidence/credible interval), ideally using structured tables or plots.	**Results** (Section 4.2), *Pages 6 – 7; Table 2*
Results of	20a	For each synthesis, briefly summarise the characteristics and risk of bias among contributing studies.	**Results** (Section 4.3),

Section and Topic	Item #	Checklist item	Location where item is reported
syntheses			*Pages 9 – 15;* **Limitations** (Section 7); *Page 21.*
	20b	Present results of all statistical syntheses conducted. If meta-analysis was done, present for each the summary estimate and its precision (e.g. confidence/credible interval) and measures of statistical heterogeneity. If comparing groups, describe the direction of the effect.	*Not applicable*
	20c	Present results of all investigations of possible causes of heterogeneity among study results.	*Not applicable*
	20d	Present results of all sensitivity analyses conducted to assess the robustness of the synthesized results.	*Not applicable*
Reporting biases	21	Present assessments of risk of bias due to missing results (arising from reporting biases) for each synthesis assessed.	**Limitations** (Section 7), *Page 21*
Certainty of evidence	22	Present assessments of certainty (or confidence) in the body of evidence for each outcome assessed.	**Certainty and Strength of Evidence** (Section 6), *Pages 20 – 21.*
**DISCUSSION**			
Discussion	23a	Provide a general interpretation of the results in the context of other evidence.	**Discussion** (Sections 5.1 & 5.2), *Pages 16 – 20*
	23b	Discuss any limitations of the evidence included in the review.	**Certainty and Strength of Evidence** (Section 6), *Pages 20 – 21.*
	23c	Discuss any limitations of the review processes used.	**Limitations** (Section 7), *Page 21*
	23d	Discuss implications of the results for practice, policy, and future research.	**Future research** (Section 9), *Pages 22 – 23*
**OTHER INFORMATION**			
Registration and protocol	24a	Provide registration information for the review, including register name and registration number, or state that the review was not registered.	**Methodology** (Section 3), *Page 6*
	24b	Indicate where the review protocol can be accessed, or state that a protocol was not prepared.	**Methodology** (Section 3), *Page 6*
	24c	Describe and explain any amendments to information provided at registration or in the protocol.	*Not applicable*
Support	25	Describe sources of financial or non-financial support for the review, and the role of the funders or sponsors in the review.	*Page 24*
Competing interests	26	Declare any competing interests of review authors.	*Page 24*
Availability of data, code and other materials	27	Report which of the following are publicly available and where they can be found: template data collection forms; data extracted from included studies; data used for all analyses; analytic code; any other materials used in the review.	*Page 24*

References

Birkle, C., Pendlebury, D. A., Schnell, J., & Adams, J. (2020). Web of Science as a data source for research on scientific and scholarly activity. *Quantitative Science Studies, 1*(1), 363–376. https://doi.org/10.1162/qss_a_00018

CASP. (n.d.). *CASP Checklists—Critical Appraisal Skills Programme.* Retrieved April 28, 2025, from https://casp-uk.net/casp-tools-checklists/

Gusenbauer, M., & Haddaway, N. R. (2020). Which academic search systems are suitable for systematic reviews or meta-analyses? Evaluating retrieval qualities of Google Scholar, PubMed, and 26 other resources. *Research Synthesis Methods, 11*(2), 181. https://doi.org/10.1002/jrsm.1378

Hong, Q. N., Pluye, P., Fabregues, S., Bartlett, G., Boardman, … & Vedel, I. (n.d.). *MIXED METHODS APPRAISAL TOOL (MMAT) VERSION 2018 User guide*.

Hong, Q. N., Pluye, P., Fàbregues, S., Bartlett, G., Boardman, F., Cargo, M., Dagenais, P., Gagnon, M.-P., Griffiths, F., Nicolau, B., O'Cathain, A., Rousseau, M.-C., & Vedel, I. (2019). Improving the content validity of the mixed methods appraisal tool: A modified e-Delphi study. *Journal of Clinical Epidemiology, 111*, 49-59.e1. https://doi.org/10.1016/j.jclinepi.2019.03.008

Hoque, M. R., Rahman, M. S., Nipa, N. J., & Hasan, M. R. (2020). Mobile health interventions in developing countries: A systematic review. *Health Informatics Journal, 26*(4), 2792–2810. https://doi.org/10.1177/1460458220937102

Indra, B., Palmasutra, V., & Setyawan, F. A. (2024). Effectiveness of Digital Interventions in Reducing Occupational Stress: A Systematic Review. *Portuguese Journal of Public Health,* 1–14. https://doi.org/10.1159/000540748

Katrak, P., Bialocerkowski, A. E., Massy-Westropp, N., Kumar, V. S., & Grimmer, K. A. (2004). A systematic review of the content of critical appraisal tools. *BMC Medical Research Methodology, 4*(1), 22. https://doi.org/10.1186/1471-2288-4-22

Long, H. A., French, D. P., & Brooks, J. M. (2020). Optimising the value of the critical appraisal skills programme (CASP) tool for quality appraisal in qualitative evidence synthesis. *Research Methods in Medicine & Health Sciences, 1*(1), 31–42. https://doi.org/10.1177/2632084320947559

Meho, L. I., & Yang, K. (2007). Impact of data sources on citation counts and rankings of LIS faculty: Web of science versus scopus and google scholar. Journal of the American *Society for Information Science and Technology, 58*(13), 2105–2125. https://doi.org/10.1002/asi.20677

Munn, Z., Moola, S., Lisy, K., Riitano, D., & Tufanaru, C. (2015). Methodological guidance for systematic reviews of observational epidemiological studies reporting prevalence and cumulative incidence data. *JBI Evidence Implementation, 13*(3), 147. https://doi.org/10.1097/XEB.0000000000000054

Ng, M. K., Yousuf, B., Bigelow, P. L., & Van Eerd, D. (2015). Effectiveness of health promotion programmes for truck drivers: A systematic review. *Health Education Journal, 74*(3), 270–286. https://doi.org/10.1177/0017896914533953

Ossom Williamson, P., & Minter, C. I. J. (2019). Exploring PubMed as a reliable resource for scholarly communications services. *Journal of the Medical Library Association: JMLA, 107*(1), 16–29. https://doi.org/10.5195/jmla.2019.433

Page, M. J., McKenzie, J. E., Bossuyt, P. M., Boutron, I., Hoffmann, T. C., Mulrow, C. D., … Moher, D. (2021a). The PRISMA 2020 statement: An updated guideline for reporting systematic reviews. *BMJ (Clinical Research Ed.), 372*, n71. https://doi.org/10.1136/bmj.n71

Page, M. J., Moher, D., Bossuyt, P. M., Boutron, I., Hoffmann, T. C., … McKenzie, J. E. (2021b). PRISMA 2020 explanation and elaboration: Updated guidance and exemplars for reporting systematic reviews. *BMJ, 372.* https://doi.org/10.1136/bmj.n160

Pernsley. (2016). *Sorting and Filtering Data with Excel—Learn Excel Now*. https://www.learnexcelnow.com/sorting-and-filtering-data-with-excel/

Richter, R. R., & Austin, T. M. (2012). Using MeSH (Medical Subject Headings) to Enhance PubMed Search Strategies for Evidence-Based Practice in Physical Therapy. *Physical Therapy, 92*(1), 124–132. https://doi.org/10.2522/ptj.20100178

Schotten, M., El Aisati, M., Meester, W. J. N., Steiginga, S., & Ross, C. A. (2017). *A Brief History of Scopus: The World's Largest Abstract and Citation Database of Scientific Literature*. In F. J. Cantú-Ortiz (Ed.), Research Analytics (1st ed., pp. 31–58). Auerbach Publications. https://doi.org/10.1201/9781315155890-3

South, A., Bailey, J., Parmar, M. K., & Vale, C. L. (2019). Effectiveness and acceptability of methods of communicating the results of clinical research to lay and professional audiences: protocol for a systematic review. *Systematic Reviews*, *8*(1), 150.

TRB. (n.d.). *TRID - Transport Research International Documentation*. Retrieved April 28, 2025, from https://trid.trb.org/

Waffenschmidt, S., Knelangen, M., Sieben, W., Bühn, S., & Pieper, D. (2019). Single screening versus conventional double screening for study selection in systematic reviews: A methodological systematic review. *BMC Medical Research Methodology, 19,* 132. https://doi.org/10.1186/s12874-019-0782-0

Wang, M., Cooper, R., & Green, D. (2023). Insomnia medication use by university students: A systematic review. *Pharmacy*, *11*(6), 171.
